# Private Selective Sweeps Identified from Next-Generation Pool-Sequencing Reveal Convergent Pathways under Selection in Two Inbred *Schistosoma mansoni* Strains

**DOI:** 10.1371/journal.pntd.0002591

**Published:** 2013-12-12

**Authors:** Julie A. J. Clément, Eve Toulza, Mathieu Gautier, Hugues Parrinello, David Roquis, Jérôme Boissier, Anne Rognon, Hélène Moné, Gabriel Mouahid, Jérôme Buard, Guillaume Mitta, Christoph Grunau

**Affiliations:** 1 Univ.Perpignan Via Domitia, Ecologie et Evolution des Interactions, UMR 5244, Perpignan, France; 2 CNRS, Ecologie et Evolution des Interactions, UMR 5244, Perpignan, France; 3 INRA, UMR CBGP (INRA – IRD – Cirad – Montpellier SupAgro), Montferrier-sur-Lez, France; 4 MGX Montpellier GenomiX, Montpellier, France; 5 CNRS, Institut de Génétique Humaine, UPR 1142, Montpellier, France; University of Melbourne, Australia

## Abstract

**Background:**

The trematode flatworms of the genus *Schistosoma*, the causative agents of schistosomiasis, are among the most prevalent parasites in humans, affecting more than 200 million people worldwide. In this study, we focused on two well-characterized strains of *S. mansoni*, to explore signatures of selection. Both strains are highly inbred and exhibit differences in life history traits, in particular in their compatibility with the intermediate host *Biomphalaria glabrata*.

**Methodology/Principal Findings:**

We performed high throughput sequencing of DNA from pools of individuals of each strain using Illumina technology and identified single nucleotide polymorphisms (SNP) and copy number variations (CNV). In total, 708,898 SNPs were identified and roughly 2,000 CNVs. The SNPs revealed low nucleotide diversity (π = 2×10^−4^) within each strain and a high differentiation level (Fst = 0.73) between them. Based on a recently developed *in-silico* approach, we further detected 12 and 19 private (*i.e.* specific non-overlapping) selective sweeps among the 121 and 151 sweeps found in total for each strain.

**Conclusions/Significance:**

Functional annotation of transcripts lying in the private selective sweeps revealed specific selection for functions related to parasitic interaction (*e.g.* cell-cell adhesion or redox reactions). Despite high differentiation between strains, we identified evolutionary convergence of genes related to proteolysis, known as a key virulence factor and a potential target of drug and vaccine development. Our data show that pool-sequencing can be used for the detection of selective sweeps in parasite populations and enables one to identify biological functions under selection.

## Author Summary

Adaptation of parasites to their environment is governed by the principle of selection. Favourable mutations are fixed in populations while deleterious mutations are progressively eliminated. Here, we aimed to find signatures of selection in two strains of *Schistosoma mansoni*, the causative agent of intestinal schistosomiasis. The strains differ in specific characters, in particular in their capacity to infect intermediate host snails. The reason for this is unknown and understanding it could help control the spreading of the disease. Finding footprints of adaptation to different snail hosts would lead to the discovery of genes that are particularly important for the interaction. Since a single parasite does not contain sufficient DNA to be sequenced, we pooled several individuals, sequenced them as a whole analysed them. In the regions under selection we found genes that are indeed linked to the parasitic lifestyle. We also discovered that natural selection led to diversification of genes that are related to proteolysis, the process by which the parasite destroys host tissue. The related proteins are considered good targets for drug development and vaccination. Our results suggest that in natural populations many variants of these genes exist and that they evolve rapidly, which might hamper therapeutic approaches.

## Introduction

In addition to their obvious importance as threats to physical and economical well-being, parasites constitute an interesting group of organisms in which to investigate adaptation and selection. Parasites closely interact with their hosts and entirely depend on them for reproduction and survival. Thus, any change in a host population, which for example decreases parasite ability to penetrate host tissue, will reciprocally select for a change in the parasite such as mechanisms favouring evasion of host resistance to infection. Such an evolutionary arms race [1], [2] has been well studied in *Schistosoma mansoni* during the interaction with its intermediate host snail [3]–[6]. *S. mansoni* is a parasitic platyhelminth infecting humans in Africa, the Arabian Peninsula, and South America. It is responsible for the most severe parasitic disease after malaria in terms of morbidity [7]–[10] killing 200,000 people (WHO Technical Report Series 912: prevention and control of schistosomiasis and soil transmitted helminthiasis (WHO, Geneva, 2002)). *S. mansoni*'s life cycle is characterized by the passage through two obligatory hosts. Parasite eggs are emitted with the faeces of the definitive human or rodent host, but can also accumulate in the liver and cause the symptoms of the disease. When the eggs in host faeces come into contact with water, free-swimming larvae (miracidia) hatch and actively seek their specific intermediate host snails. After active penetration through the tegument, the parasite develops via a primary (mother) sporocyst, then daughter sporocysts releasing the cercariae that infect the vertebrate host. Then, sexual differentiation takes place within this definitive host and the mating of male and female worms leads to new egg production.

In natural populations, snail/schistosome combinations present different levels of compatibility (*i.e.* the ability for the parasite to penetrate and develop in the host) [3], [4]. Previous comparative approaches between a Brazilian (BRE) and Guadeloupean (GH2) *S. mansoni* strains showed that while the first is compatible with a sympatric *Biomphalaria glabrata* strain from Brazil, the latter is much less compatible [7]. Compatibility levels of these strains (and several others not presented in this study) are stable after several years of maintenance under laboratory conditions (Supplementary figure S1). This particular feature has made it possible to elucidate partially the molecular basis of the compatibility polymorphism at the global proteomic [3], [4], [7]–[11] and epigenetic scales [12]. In addition, these strains present significant differences in several life history traits, such as chronobiology in cercarial emission [11] and number of mother sporocysts [7]. They are therefore ideal models to investigate signatures of selection at the whole genome scale in *S. mansoni* and to elucidate the genetic basis of phenotypic variation for this parasite.

Three major classes of polymorphisms are responsible for variations in the genotype: (i) single nucleotide polymorphisms (SNPs), a modification of the nucleotide information at a single position, (ii) insertions and deletions (indels) and (iii) structural polymorphisms such as copy number variation (CNV), resulting from tandem duplications of genome segments. These variations can produce by chance favourable, neutral or deleterious phenotypic variations leading to greater, equal or lower fitness, respectively. With the recent advent of Next Generation Sequencing (NGS), hundreds of complete eukaryotic genomes are now available together with huge amounts of data on gene expression and genomic polymorphisms. Standard methods from population genetics to detect selection [3], [4], [7], [13], [14] can theoretically be applied to large-scale genomic data, but improvements have been needed to take into account the SNP ascertainment process [15].

These improved methods have been successfully applied to answer different biological questions dealing with the adaptive process, such as biological invasions [11], [16], gene selection in human populations [7], [17], and domestication [18], [19]. An obstacle for small organisms is the amount of DNA required for library generation and sequencing. In this context, pool-sequencing provides an alternative. The method consists of DNA extraction from a large number of individuals from a population (pool) followed by massive sequencing. SNP frequencies and distributions are then extracted from the sequencing data. While in principle straightforward, current methods for the detection of selective sweeps from pooled sequence SNPs had to be adapted. We used pool-HMM [20]–[22] in the current work. In this statistical method, the inference of selective sweeps (*i.e.* the elimination of standing variation in regions linked to a recently fixed beneficial mutation) is based on the allele frequency spectrum (AFS) assessed in a sliding window along each chromosome. Our approach allowed sequencing a sufficient number of individuals at moderate costs and to apply population genetics approaches without affecting population genetics estimators [23].

CNV detection is often performed by microarray-based methods comparing two genomes [1] but recent developments such as CNV-seq [24] have extended the method to NGS data. It takes advantage of the variation in the number of short reads aligned in a sliding window along each chromosome to assess CNV at the whole genome scale. As differences in CNV within the same species have been recognized to be involved in adaptive evolution [25], [26], we used NGS data to investigate the proportion of this type of variation in our comparative approach.

In the case of *S. mansoni*, we benefit from the availability of a genome assembly [27], facilitating the use of NGS-based strategy for the detection of regions under selection. In this work, we have characterized SNPs from whole genome pool-sequencing data and described their distribution before applying the new population genomics method of Boitard *et al.* [21], [22] for detecting selective sweeps as signatures of past selection in these two populations. We also describe differences in copy number variations (CNV) between the two populations as another signature of selection. We finally investigated functional aspects of all genomic regions corresponding to either private selective sweeps or structural variation and proposed evolutionary and ecological interpretations to these genetic differences found between and within BRE and GH2 strains at the whole genome scale.

## Materials and Methods

### Ethics statement

We adhered to national ethical standards established in the writ of February 1^st^, 2013 (NOR : AGRG1238753A) setting the conditions for approval, planning and operation of establishments, breeders and suppliers of animals used for scientific purposes and controls. The Ministère de l'Agriculture et de la Pêche and Ministère de l'Education Nationale de la Recherche et de la Technologie provided permit A 66040 to our laboratory for experiments on animals and certificate for animal experimentation (authorization 007083, decree 87–848) for the experimenters. Housing, breeding and animal care followed national ethical standards.

### Microsatellite analysis

DNA of twenty individual worms (10 males and 10 females) of each strain was individually extracted and genotyped using 15 microsatellite markers. Methods for DNA extraction and microsatellite amplifications were previously published [28].

### *Schistosoma mansoni* DNA preparation for high throughput sequencing

Two *Schistosoma mansoni* strains, one collected in Brazil (BRE) and the other in Guadeloupe (GH2) were used in this study. Each strain was maintained in its sympatric intermediate host (the mollusk *Biomphalaria glabrata*) and in the mouse (*Mus musculus*) or the hamster (*Mesocricetus auratus*) as a definitive vertebrate host. Genomic DNA was isolated from a pool of a hundred adult individuals. Parasite tissues were digested with 300 mg/L Protease K (Merck, Darmstadt, Germany) in 20 mM TRIS pH 8; 1 mM EDTA; 100 mM NaCl; 0.5% SDS at 55°C overnight. DNA was extracted by two successive rounds of phenol/chloroform followed by chloroform extraction. Precipitation of DNA was done by adding an equal volume of isopropanol/sodium acetate at room temperature [29] and DNA was collected by centrifugation at 12000 rpm for 30 min. After washing with 1 mL of 70% ethanol and air drying, DNA was dissolved in 200 µL of ultrapure water. Quality control and quantification were performed using a spectrophotometer (BioPhotometer, Eppendorf AG, Hamburg, Germany). Twenty µg of DNA was sent to the Montpellier (France) GenomiX facility for Illumina sequencing. Both pooled DNA samples thus represented a random sample from BRE and GH2 populations. If not otherwise stated, reagents were purchased from Sigma-Aldrich (St. Louis, USA).

### Illumina (pool-) sequencing

#### Illumina library production

The Illumina TruSeq DNA sample preparation kit (FC-121-2001, Illumina Inc., San Diego, USA) was used according to the manufacturer's six-steps protocol: 1) 1 µg of DNA was sonicated for 20 minutes by using a Bioruptor (power set on “High”, cycles of 30 seconds “ON” and 30 seconds “OFF”); 2) Extremities of sonicated DNA fragments were repaired; 3) an A-base was added to these extremities; 4) Universal (5′-AATGATACGGCGACCACCGAGATCTACACTCTTTCCCTACACGACGCTCTTCCGATCT-3′) and strain-specific (C: 5′-GATCGGAAGAGCACACGTCTGAACTCCAGTCACTGACCAATCTCGTATGCCGTCTTCTGCTTG-3′ ; IC: 5′-GATCGGAAGAGCACACGTCTGAACTCCAGTCACCGATGTATCTCGTATGCCGTCTTCTGCTTG-3′) adapters were then ligated to these extremities ; 5) DNA products were migrated on 2% agarose gel and 500 bp+/−25 fragments were purified (DNA insert size of 380 bp+/−25); 6) 12 cycles of PCR amplification were performed to select only DNA fragments carrying both universal and strand specific adapters; 6) Libraries were then validated and quantified on a DNA1000 chip on a Bioanalyzer (Agilent) to determine size and concentration.

#### Illumina library clustering and sequencing conditions

The cluster generation process was performed on cBot (Illumina Inc.) by using the Illumina Paired-End DNA sample preparation kit (FC-102-1001, Illumina Inc.). DNA libraries were denatured using 0.1 N NaOH then diluted to 8 pM. Diluted libraries were first hybridized to the flow cell (one library per lane) and then amplified leading to DNA cluster formation. After DNA linearization and free-extremity blocking, the first sequencing primer was hybridized to the free-extremity of DNA fragments. Both *S. mansoni* strains were paired-end sequenced on the HiSeq 2000 (Illumina Inc.), using the SBS (Sequence By Synthesis) technique. Paired-end sequencing consisted of sequencing of the first extremity of DNA fragment, followed by the reversal of DNA molecules before the sequencing of the second extremity. Fluorescence was acquired by a camera and image analysis was performed by using HiSeq Control Software (Illumina Inc.). Base calling was achieved by using the RTA software (Illumina Inc.), which corrected and transformed the optical signal from a nucleotidic base. Reads of 100 bp from both sides of the fragments were thus obtained after this step.

### *In silico* SNP identification

If not otherwise stated, software was used with default parameters. The Fastx-toolkit version 0.0.13 (http://hannonlab.cshl.edu/fastx_toolkit/index.html) was used for quality control and initial cleaning of the sequencing reads. Adaptators were first removed and the three last bases were trimmed from reads because they showed global poor quality scores (Phred quality score <24) in most reads. We then filtered reads by their quality score and retained only reads for which at least 90% of the bases had a minimum Phred quality score of 24 (corresponding to less than 1 incorrect base call in 100 and more than 99% of base call accuracy). Paired-end data were then considered as two subsets of single-end data. Reads were aligned onto the reference genome version 5.2 [27] with the Bowtie software version 0.12.7 [30]. Because 47% of the *S. mansoni* genome consists of repeated sequences [31] we did not allow for alignment of reads that matched more than one time to avoid false positives in subsequent SNP calling. We allowed 2 mismatches and used the “best” and “strata” options. SNP calling was done with Freebayes software version 0.9.5 (https://github.com/ekg/freebayes) with parameters –min-coverage –min-alternate-fraction –min-alternate-total –pooled –ploidy 20. We tested a minimum coverage of 10 or 20 reads per SNP position (–min-coverage 10 or 20) with a minimum of 2 reads supporting the variant allele (–min-alternate-total 2) and with a minimum frequency of variant of 0.02 (–min-alternate-fraction 0.02). By retaining only SNP that met these conditions, we prevented mistakes in SNP discovery due to errors during base calling and we allowed the discovery of rare variants (up to a frequency of 0.02). Among all SNPs, we identified rare and frequent SNPs by using a 20% threshold as proposed in other studies [17], [32]. *In silico* verification of SNP calls was performed by choosing at random 20 SNPs per chromosome and per strain for each minimum coverage value 10 and 20, corresponding to a total of 640 SNPs. We visually checked the number of reads, the number of variant allele and the alternative base identity with the Next Generation Sequence Assembly Visualization software Tablet [33]. All further analyses were done on the dataset obtained with the option –min-coverage 20.

### *In vitro* SNP verification

To confirm SNP calling, we PCR amplified (primers in Table S8) and re-sequenced 14 DNA fragments covering a total of 22 SNPs. We used a specific melting temperature for each primer pair (Table S8). Sequencing was performed for each PCR product in forward and reverse directions by the GATC biotech AG Company (Konstanz, Germany) using Sanger technology. Sequences were aligned on the reference sequence with Sequencher software version 4.5 (Gene Codes Corporation, Ann Arbor, USA) and SNPs were visually confirmed.

### Structural variants

We used CNV-seq to detect copy number variation [24] using a 10^−6^ p-value threshold. Briefly, this algorithm uses variation in sequence coverage in a sliding window between genomic reads mapped to a reference genome to detect variations in sequence copy numbers. Plots were drawn using the associated cnv package in R. We manually checked protein-coding genes contained in the 20 largest genomic regions and the 20 regions with the highest difference in copy number between the BRE and GH2 strain by using the coordinates of the regions in the *Schistosoma mansoni* local Gbrowse instance of the genome (http://genome.univ-perp.fr).

### Characterization of genetic variability

We used standard tools available on a local Galaxy instance [34] to extract information from the pool-sequencing data. Samtools version 0.1.18 [33] was used to produce mpileup files with read coverage information and call quality from the alignment BAM files. Nucleotide diversity was evaluated using the unbiased Tajima's Pi and Watterson Theta estimators proposed by Futschik and Schlotterer [35] and implemented in Popoolation [36]. Briefly, along each chromosome, each estimator was computed in 50 kb sliding windows (with an overlap of 25 kb between consecutive windows) with the following additional options –min-count 2 –min-coverage 4 –max-coverage 400 –min-qual 20. Considering smaller (10 kb) or larger (100 kb) window size did not affect the results. Fixation index (*Fst*) was assessed on SNPs by using Popoolation2 [37]. We considered all SNPs for which at least 6 reads supported the minor allele for both population simultaneously (–min-count 6), and with a coverage ranging between 20 and 200 reads (–min-coverage 20 ; –max-coverage 200). Mean fixation index between BRE and GH2 was calculated as the average of all *Fst* values obtained for individual SNPs.

### Whole-genome scan for footprints of selection

In the current version of the genome, the Z and the W chromosomes are put into a single linkage group [27] despite their physical separation into two chromosomes. SNPs could therefore reflect differences between Z and W and not between target and reference genome. For this reason we excluded them from further analyses of selection. To identify footprints of selection on the 7 autosomes of *Schistosoma mansoni* from BRE and GH2 pool sequences, we relied on the approach recently proposed by Boitard *et al.* [21] implemented in the pool_hmm program [22]. The following option were used to run the analyses:-C 1000 -k 1e-10 –pred -t *θ_w_*, where *θ_w_* corresponds to the average unbiased Watterson theta estimator of nucleotide diversity for the population of interest (see above). This method relies on the study of the allele frequency spectrum (AFS) within sliding windows along the genomic sequences. The AFS is expected to be distorted in regions subjected to selection. The model allows estimating the probability of each SNP to belong to one of the three possible (hidden) states (i) neutral, (ii) intermediate and (iii) selected. In practice, one of the critical parameter of the model (defined with the –k option) is the transition probability *q* assumed between states. The larger the *q*, the less evidence is required for transition to selection and the more sweep candidates will be detected. In our analyses, we used *q* = 10^−10^ but also tested less stringent values (*q* = 10^−9^). As shown in Table S9 and as expected, this leads only to a slightly higher number of footprints. We identified overlapping and private selective sweeps between the two strains by comparing their genomic position for each chromosome. As some genomic region swept in one strain could overlap several shorter regions swept in the other strain (and *vice versa*), we counted overlapping regions as the exact number of genome fragments really overlapping (*i.e.* counting the number of shorter instead of counting only the larger region covering them). Private selective sweeps of each strain correspond to all genomic regions that strictly did not overlap any sweeps of the other strain. Identification of known transcripts in private selective sweeps for both strains was performed using the genome coordinates of the regions and the *Schistosoma mansoni* local Gbrowse instance of the genome. Confidence index was calculated for each specific selective sweep as the maximum of −log(1-*q_i_*) over the window, where *q_i_* is the posterior probability of hidden state “Selection” given after simulations.

### Functional characterization of selected genes

To characterize the molecular functions of the transcripts contained in selective sweeps, we first performed functional annotations of a recent *S. mansoni* transcriptome (unpublished results) by using the Blast2GO software [38]. After the blast step, we mapped gene ontology (GO) terms (BRE: NodeScore = 10, alpha = 0.4; GH2: NodeScore = 15, alpha = 0.2). We then scanned the proteins with Interproscan [39] and performed GO-Enzyme code mapping to improve annotations before running the annotation step. We finally merged results from these three annotation methods before making functional analyses. We checked if our lists of transcripts matched with proteins that were identified in an earlier study comparing these two strains at the proteomic level [3] using tblastn on the 5.2 version of the *S. mansoni* genome. Results were verified by visual inspection on a local GBrowse instance of the genome and transcriptome. We then performed functional analyses with Blast2GO tools for the list of transcripts from selective sweeps but not for the list of protein-coding genes from CNV data because this latter list was not exhaustive (see the “Structural variants” previous section). Enrichment analysis was performed for each strain by using the Fisher's exact test with the P-value filter mode set at the default value of 0.05 (Bonferroni correction is applied). Combined graphs were then drawn for each strain from two kinds of data: i) the total list of transcripts found in selective sweeps and ii) the reduced list of transcripts obtained after enrichment analysis. In both cases, Score alpha and Seq Filter value were set at the default values of 0.6 and 5 respectively. Graphs displaying process or function were built on the node score criterion set at the value of 10 or 15 depending on the graph complexity obtained. We also compared molecular pathways in which selection was found by building KEGG maps for each strain within the Blast2GO application.

### Synonymous vs. non-synonymous SNPs

SNPEff was used to scan synonymous and non-synonymous SNPs in exons of the whole genome of our parasite model. We downloaded the GFF3 file for the latest assembly (v5.2, nov. 2011) from the Sanger centre (ftp://ftp.sanger.ac.uk/pub/pathogens/Schistosoma/mansoni/genome/GFF/Smansoni_gff_21032012.tar.gz) and modified it using custom scripts so that gff data and fasta data were separated. We then built a SnpEff database using snpEff.jar with “build -gff3”. A total of 13,385 genes and 14,395 transcripts were detected. The snpEff.config file was modified accordingly and snpEff run with the following parameters: -c snpEff.config -i vcf -o txt -upDownStreamLen 5000 -no None –stats.

### Statistical analyses

If not otherwise mentioned, statistical analyses were done on R version 2.15.1 [40]. To test if the SNP density was correlated to the chromosome or the strain, we constructed linear models and tested for the significance of parameters (p>0.05) by analyses of variance (ANOVAs).

### Data availability

Sequencing reads are available at the NCBI sequence read archive under study accession number SRP016500 (alias PRJNA177787). Freebayes output (SNPs) is available in vcf and mpileup format at http://2ei.univ-perp.fr/?page_id=2007 and http://methdb.univ-perp.fr/downloads/.

## Results

### Choice of strains

Since pool-sequencing is not yet an established technique for detection of selection we reasoned that it would be suitable to use strains with presumably low genetic diversity. We had earlier characterized the proteome of two laboratory strains of *S. mansoni* of different geographic origins [41] and we had characterized their life history traits such as host compatibility in detail [42]. A comparison of the epigenomes of both strains had identified differences in chromatin structure in several loci, among them a metalloprotease of the neutral endopeptidase (NEP) family potentially involved in immuno-modulation of *B. glabrata* [12]. A preliminary assessment of genetic variation in both strains was performed by sequencing 15 microsatellites previously described [28]. All microsatellites markers of the Brazilian (BRE) strain were fixed and homozygous. In the Guadeloupean (GH2) strain only one marker (SMDO11) showed two distinct genotypes. Among the 14 fixed markers in the two strains, 6 are shared by BRE and GH2. We concluded that both strains were sufficiently genetically homogenous to perform massive sequencing of a population and phenotypically sufficiently well characterized to potentially link sequence variations to phenotypic traits.

### Pool-sequencing

Sequencing of BRE and GH2 strains produced roughly 500,000,000 raw clusters each (Table S1). About 50% passed quality checking (Table S1) and were subsequently aligned to the unique sequences of the reference genome (strain NMRI). Unsurprisingly, we successfully aligned only 46.95% and 59.29% of high-quality reads for BRE and GH2 strains, respectively. This is consistent with the large proportion (47%) of repetitive sequences in the *S. mansoni* genome [31]. Sequencing data are available under study accession number SRP016500 (alias PRJNA177787) at the NCBI sequence read archive.

#### SNP description

After read alignment on the reference genome and SNP calling for each strain separately, we identified 672,467 polymorphic sites for GH2 and 464,746 for BRE. About 0.2% of them were multiple alleles (*i.e.* more than one alternative allele to the reference genome). Density of polymorphic sites was thus 1.35/kb for BRE (1 polymorphism every 738 bp on average) and 1.96/kb (1 polymorphism every 510 bp on average) for GH2. For all subsequent analyses, we only focused on polymorphic sites that were localized on the annotated and linkage-group/chromosome-level assembled parts of the genome. For the present work, we define SNP at the species level as a nucleotide that is variable within the *S. mansoni*'s genome (*i.e.* with at least one alternative allele different from the reference genome) in at least one of the studied populations/strains or individuals. A SNP can thus fall into one of these two following categories: (i) Private SNP, that is a variable nucleotide within a unique population and fixed to the reference allele in other populations and (ii) Shared SNP, a nucleotide which is different from the reference in at least two populations. Alternative allele frequency (AAF) of a private SNP can thus be equal to zero when fixed in the population, but can take all values from zero (excluded) to one, if several alleles for this position are present in the population. Based on this definition, we found a total of 708,898 SNPs, among which 65,490 were shared (9.2%), 253,321 were private to BRE and 390,087 private to GH2 (Table 1). SNPs were also classified in two categories (rare and frequent) based on the AAF with a cut-off value of 0.2 as was previously proposed [17]. We also defined a sub-category of fixed alternative allele (AAF equal to one) within the “frequent” category. The majority of shared SNPs (70.9%) were fixed (Table 1) or frequent, while private SNPs belonged mainly to the rare category with an AFF ≤0.2 (Table 1). For both shared and private SNPs, the majority of SNPs that were classified as frequent (almost 90% in each case) corresponded to fixed alleles (Table 1).

**Table 1:** Distribution of polymorphic sites and single-nucleotide polymorphism (SNP) for Brazilian (BRE) and Guadeloupean (GH2) strains of *Schistosoma mansoni* used in this study.

	BRE	GH2
1 - Total number of polymorphic sites	464,746	672,467
1.1 - Polybase substitution (% of total polymorphic sites)	1,044 (0.2%)	1,412 (0.2%)
2 - Total SNPs located on chromosomes (autosomes + ZW)	708,898 (568,046+140,852)	
2.1 - Shared SNPs (% of total SNP)	65,490 (9.2%)	
2.1.1 - Rare (% of total SNP - % of total shared)	9,744 (1.4%–20.5%)	
2.1.2 - Frequent: (% of total SNP - % of total shared)	55,746 (7.8%–79.5%)	
2.1.2.1 - Fixed (% of total SNP - % of total shared)	46,440 (6.6%–70.9%)	
2.2 - Private SNPs (% of total SNP)	253,321 (35.8%)	390,087 (55.0%)
2.2.1 - Rare (% of total SNP - % of total private)	173,336 (24.5%–68.4%)	283,456 (40.0%–72.7%)
2.2.2 - Frequent: (% of total SNP - % of total private)	79,985 (11.3%–31.6%)	106,631 (15.0%–27.3%)
2.2.2.1 - Fixed (% of total SNP - % of total private)	69,620 (9.8%–27.5%)	94,666 (13.4%–24.3%)

AAF = alternative allele frequency. Rare SNP correspond to 0<AAF < = 0.2, frequent correspond to AAF>0.2 and fixed correspond to AAF = 1.

#### Between chromosome distribution

SNP number on each chromosome for both strains (bars in Figure S2) was highly correlated to chromosome length (r = 0.93, p<0.0001) but SNP density (dots in Figure S1) significantly differed between chromosomes (F_7,7_ = 15.76, p<0.001) and between strains (F_1,7_ = 346.28, P<0.001). However, no differences in SNP density repartition between strains were found as SNP densities are highly correlated (rho = 0.97, p<0.001).

#### Within chromosome distribution

SNPs were not equally distributed within chromosomes as we observed SNP clustering for each chromosome of each strain, *i.e.* SNPs tend to aggregate preferentially in some regions and to be lacking in others (Figure S3). This non-random distribution of SNPs across the genome was previously observed in other models [43], [44] and is described here for the first time for a metazoan parasite.

Moreover, the distribution of SNP clusters seemed at least partly different between BRE and GH2 strains. Some large peaks of SNP number were common to both strains (see around 55,640,000 window position on ZW sex chromosomes linkage-group, Figure S3H) while others were specific to BRE (see around 3,960,000 window position on Chromosome 6, Figure S3F) or to GH2 (see around 1,140,000 window position on Chromosome 7, Figure S3G).

#### Nucleotide diversity

We excluded sex chromosomes from subsequent analysis based on allele frequency because of the impossibility to assign SNPs specifically to Z or W chromosomes due to their assemblage into a single linkage group in the present version of the genome. Using Popoolation [36] on the 568,046 SNPs located on autosomes only, we estimated nucleotide diversity. Diversity levels are low with Tajima's Pi (and Watterson Theta) indexes of 2.10^−4^ (2.9.10^−4^) for GH2 and 1.8.10^−4^ (2.6.10^−4^) for BRE (Table S2 for full details of diversity measures). This corresponds to an effective population size of hundreds to thousands individuals, assuming a mutation rate around 10^−7^–10^−8^ typically found in other eukaryotic genomes [45], [46]. This population size is consistent with our laboratory conditions in which vertebrate hosts are usually infected with 140 (mice) to 400 (hamster) cercariae.

#### Population differentiation

Based on the mean fixation index value (*Fst* = 0.73±0.41 sd), BRE and GH2 populations were highly differentiated. More precisely, among the 229,044 SNPs meeting criteria used in this analysis (see Material & Methods section), 143,391 (60.6%) are fixed in one of the two populations (*Fst* = 1).

#### Identification of structural variants

**Figure 1:**
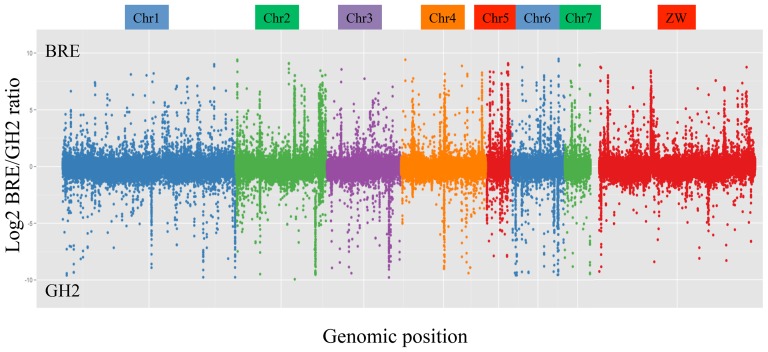
Log 2 ratio plot of copy number variations (BRE/GH2) along the *Schistosoma mansoni* genome. The x-axis represents the genome position in basepairs. Chromosomes are colour coded. The y-axis shows log2 ratio between BRE and GH2. Positive values indicate overrepresentation of a region in BRE, negative values indicate overrepresentation of a region in GH2.

We used CNV-seq (R version 2.10.1, package ‘cnv’ version 0.2–7.) to detect copy number variations between the two populations [24]. Because our sequences do not come from a single individual but a population, we used a stringent p-value of 10^−6^ to decrease background variation in coverage. We identified 1,474 CNV for autosomes and 529 for W sex chromosome linkage group with a calculated sliding window of 1,554 and 1,726 nucleotides, respectively (Table 2, Figure 1, Table S3). This number of CNVs is probably underestimated due to relatively modest average sequencing depth. We also analysed independently unassembled scaffolds larger than 500 kb. As outlined above, reads that matched the reference genome more than one time were removed before SNP calling. Manual check of regions with CNV confirmed that they correspond to genomic regions containing single-copied protein coding genes (Supplementary Table S3). We are thus confident that CNV that have been detected in this work do not correspond to repeated sequences.

**Table 2:** CNV characteristics for autosomes and ZW-linkage group of *Schistosoma mansoni*.

	Optimal window size used by CNV-seq (bp)	Percentage of CNV (%)	CNV total nucleotide content	CNV count	Mean size (bp)	Median size (bp)	Min-max size (bp)
Autosomes	1,554	4.6%	9,236,932	1,474	6,267	18,649	3,107–87,025
W linkage group	1,726	6.7%	4,010,361	529	7,581	15,534	3,451–268,393

### Identification of selective sweeps

**Figure 2:**
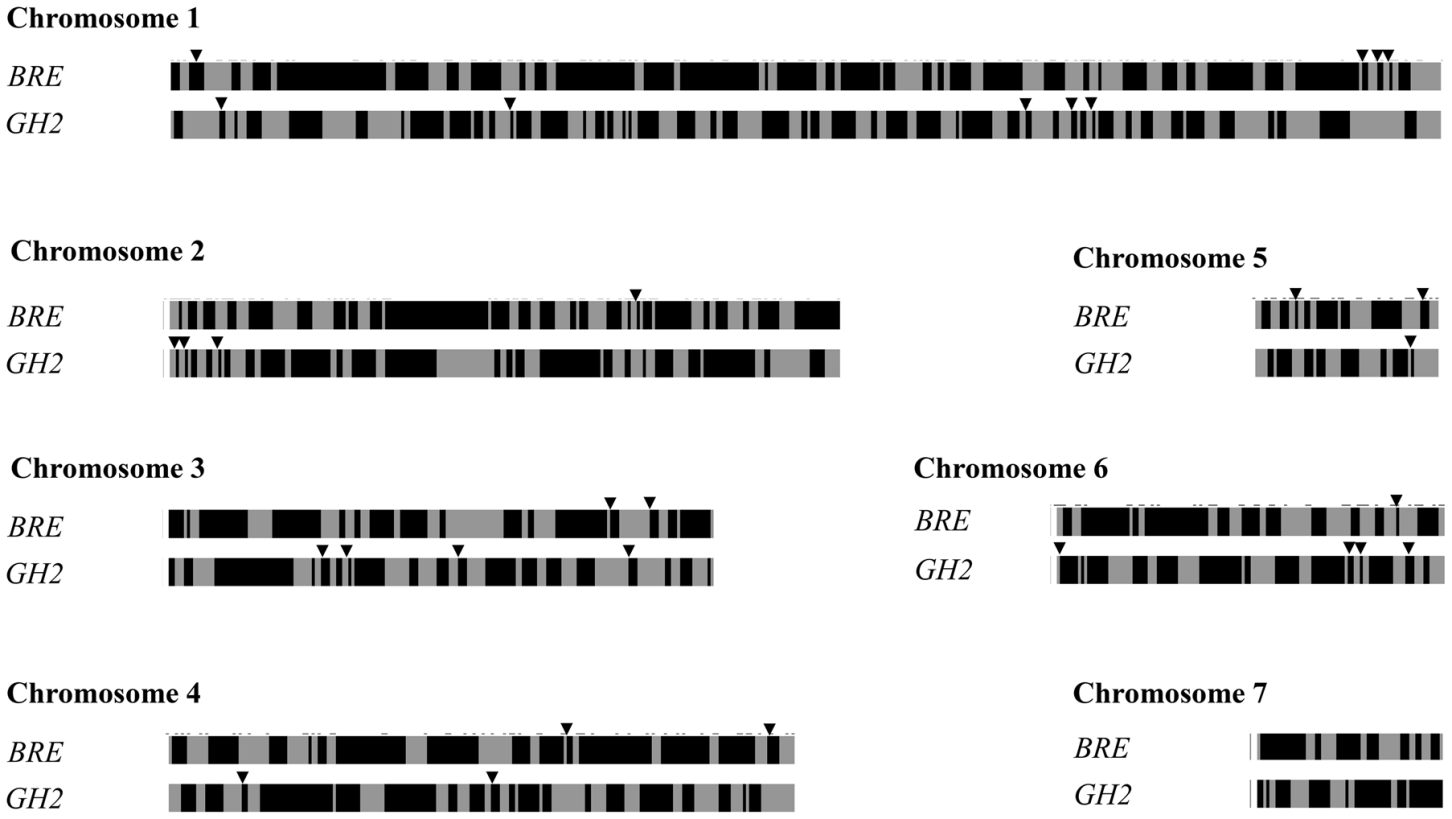
Chromosomal representation of selective sweeps (in black) and non-swept regions (grey) over the 7 autosomes of *Schistosoma mansoni* for BRE and GH2. Black arrowheads indicate the non-overlapping specific selective sweeps for both strains.

We used the pool-HMM method [20]–[22] to detect selective sweeps from pool-sequencing data. The question of selective sweeps on sex chromosomes was not addressed in this study. We detected a total of 121 and 151 selective sweeps across the 7 autosomes for BRE and GH2, respectively (Figure 2). We counted a total of 146 overlapping regions, and identified 12 and 19 private selective sweeps for BRE and GH2, respectively (Figure 2) which were differently distributed along the 7 chromosomes (Table 3). Most of these private selective sweeps spanned 100 kb to 1 Mb which corresponded to larger regions than those identified with this method for the X chromosome of *Drosophila melanogaster* [21].

**Table 3:** Selective sweep regions and number of corresponding genes for (A) BRE and (B) GH2 strains.

A	Start	End	Length	Number of genes	CI
Chr1	1,194,541	2,357,392	1,162,852	56	Inf
	61,387,283	62,009,343	622,061	23	11.15
	62,218,658	62,721,763	503,106	19	6.67
	62,877,538	63,098,318	220,781	9	2.71
Chr2	24,145,118	24,315,462	170,345	6	2.83
Chr3	22,823,405	23,467,278	643,874	35	Inf
	24,930,649	25,380,787	450,139	14	Inf
Chr4	20,500,484	21,005,970	505,487	24	8.63
	31,100,629	31,804,241	703,613	36	Inf
Chr5	2,117,276	2,386,209	268,934	17	7.53
	8,814,688	9,017,158	202,471	12	9.05
Chr6	17,508,128	17,649,604	141,477	6	3.94
Chr7	-	-	-	-	-

B	Start	End	Length	Number of genes	CI
Chr1	2,509,484	3,001,529	492,046	43	12.00
	17,514,351	17,728,884	214,534	16	6.36
	44,140,447	44,504,258	363,812	15	4.70
	46,537,352	46,758,483	221,132	13	4.98
	47,463,874	47,701,141	237,268	7	2.83
Chr2	91	101,151	101,061	3	4.07
	832,591	953,172	120,582	8	4.24
	2,627,180	2,753,105	125,926	6	4.74
Chr3	7,872,267	8,528,892	656,626	24	Inf
	9,155,168	9,454,868	299,701	11	11.10
	15,054,008	15,663,195	609,188	52	10.00
	23,728,696	24,321,481	592,786	29	9.51
Chr4	3,816,163	4,152,576	336,414	12	Inf
	16,762,042	17,264,224	502,183	23	Inf
Chr5	7,988,578	8,174,020	185,443	9	3.64
Chr6	18	139,169	139,152	8	9.74
	11,873,433	13,026,934	1,153,502	54	Inf
	15,736,118	15,951,459	215,342	14	3.29
	18,289,818	18,553,369	263,552	21	10.55
Chr7	-	-	-	-	-

Confidence Index (CI) was calculated as the maximum of −log(1-*q*) over the window, where *q* is the posterior probability of hidden state “Selection”. (Inf. = infiny).

### Functional annotation and analyses

#### Structural variants

We manually checked the CNV encompassing the twenty largest chromosomal regions for the presence of protein-coding genes (Table S4). The largest duplicated region in linkage-group ZW correspond to an additional 268 kb in BRE (Figure 1, Figure S4) and contains 8 protein-coding genes (nucleolar protein 56, histidine triad nucleotide binding protein 1, transcription factor 7 2, exosome complex component RRP45, spermatogenesis associated protein 6, junctophilin 2 and two genes encoding hypothetical proteins). The largest duplicated region in autosomes corresponds to an additional 87 kb on chromosome 3 in GH2 (Figure 1, Figure S4) and contains 2 protein-coding genes encoding telomerase components. We also checked the CNV encompassing the twenty chromosomal regions with the highest value of log2 ratio (*i.e.* with the largest differences in copy number between BRE and GH2) (Table S5). Twelve corresponded to regions without protein-coding genes, four corresponded to regions with genes encoding hypothetical proteins and three contained known genes. Particularly, we identified the presence of (i) a member of immunophilins, a large group of proteins with peptidyl prolyl-isomerase activity (PPI-ase) that exhibit high specificity in binding to immunosuppressive agents (such as cyclosporine); (ii) a homolog of SPARC (secreted protein acidic and rich in cysteine), a secreted glycoprotein involved in interactions between cells and extracellular matrix [47], both with a higher copy numbers in BRE, and (iii) a M13 family peptidase (that present more copies in GH2), a type II membrane metallo-endopeptidase acting on extracellular substrates [48], belonging to a family of proteases for which another member was identified to be epigenetically different between the two strains [12]. Among the 40 CNV regions checked, one was found in both the Top20 (by size) and in the Top20 (by log2 ratio) (CNVR_270) (Table S4 and S5). It contains the abovementioned M13 family proteases.

#### Private selective sweeps

To identify which functions and/or molecular pathways could have been adaptively selected and could thus be responsible for differences in life history traits and proteomes, we here focused on private selective sweeps of each strain (under arrows in Figure 2, Table 3) assuming that shared selective sweeps could be due to the common laboratory environment.

Within the 12 BRE and 19 GH2 private selective sweeps, we found a total of 592 and 791 predicted genes respectively (Table S5A and Table S5B). Visual inspection of both lists of transcripts in regions under selection revealed the presence of different peptidases for BRE and GH2. In particular, we identified several Cathepsin D and Cathepsin B-like peptidases (Table S6) in BRE, two proteins involved in parasitic activity [49], [50].

In a previous work [41], proteomes of sporocysts of *S. mansoni* BRE and GH2 strains had been compared and 17 differentially expressed proteins and/or protein isoforms were revealed . We checked if genes encoding these proteins were located in the regions under selection that we identified in this work. Nine of these proteins (S.mMucin-like, now SmPoMuc family) are encoded by members of a gene family and were already characterized in more detail [51]. Since duplicated loci such as these were excluded in the short-read alignment process, these genes were excluded from the present analysis. None of the 7 genes coding for the 8 remaining protein isoforms are located in any region of private selective sweeps either.

Comparison of combined graphs from Blast2GO gene ontology analysis highlighted several biological processes (Supplementary figure S5). Proteolysis and transport appeared as common biological processes selected in each of the two strains, despite the fact that they correspond to non-overlapping lists of transcripts found in private selective sweeps. Specific processes were also identified, such as cell-cell adhesion for the BRE strain or redox reactions and protein phosphorylation for the GH2 strain.

Using the Kyoto Encyclopaedia of Genes and Genomes (KEGG) database, we looked for molecular pathways containing enzymes appearing in regions under selection for each strain. We identified 26 and 34 enzymes (distributed over 24 and 22 pathways) for BRE and GH2, respectively (Table S7). We found five common pathways (Purine metabolism, Riboflavin metabolism, Butanoate metabolism, Aminobenzoate degradation and Carbon fixation). Two of them (Riboflavin metabolism and Aminobenzoate degradation) were highlighted by a unique common enzyme (Enzyme code 3.1.3.2 corresponding to a phosphatase) found under selection and present in both pathways. The three other common pathways were highlighted by enzymes differing between BRE and GH2. In specific pathways, we noted the presence of the N-Glycan biosynthesis for BRE (Table S7), involved in the glycosylation of proteins. Differential glycosylation is involved in the generation of SmPoMucs variants and in part is responsible for compatibility with the intermediate host [42], [51].

### Synonymous vs. non-synonymous SNPs

Along autosomes, we identified 10,982 and 14,811 SNPs in exons for BRE and GH2 respectively. Proportions of non-synonymous (NonSyn-) and synonymous (Syn-) SNPs within and outside swept regions were similarly distributed for both strains (Supplementary figure S6). Interestingly, 49% of them were Non-Syn-SNPs and found within selective sweeps. We also found more than three times more Non-SynSNPs than Syn-SNPs in private selective sweeps.

## Discussion

In this study we have sequenced the genomes of two *Schistosoma mansoni* populations, BRE and GH2, originating from Brazil and Guadeloupe, respectively. Next generation pool-sequencing was applied to this metazoan parasite to analyse genomic variations (SNP and CNV) and to scan for selective sweeps. By mapping genome reads of these two strains to the *S. mansoni* NMRI strain reference genome [27], [52], we discovered hundreds of thousands of SNPs and used these data to make the first SNP map for *S. mansoni* at the whole genome scale. We made these data available for further analyses.

Although both strains have been maintained in the lab for more than thirty years (corresponding to approximately one hundred full life-cycles), life history traits such as compatibility levels with the intermediate host as well as chronobiology were maintained overtime (Supplementary figure S1) [7], [11]. Earlier investigations had concluded that populations maintained in the laboratory rapidly decreased in diversity to become monomorphic based on the analyses of nine neutral microsatellite markers [28]. It was also previously demonstrated that the diversity of *S. mansoni* decreased dramatically after the first life cycles of laboratory maintenance, based on the analyses of 15 microsatellite markers [28]. Our global comparative approach of BRE and GH2 strains was thus fully justified by their high level of inbreeding and their well-described compatibility polymorphism at the phenotypic and molecular scales [3], [7], [12], [41], [51], [53]. The use of pool-sequencing allowed for evaluating the genome-wide diversity (SNP distribution and density, SNP frequencies and copy number variations) for each population. Intra-population diversity was low (confirming the results of the earlier microsatellite study) based on the whole-genome nucleotide diversity indexes Tajima's Pi and Watterson Theta and compared to other studies considering nucleotide diversity at this scale [54], [55]. Moreover, several lines of evidence indicate a high divergence between populations: (i) we found a high number (and thus a high density) of single-base polymorphisms for both strains when compared to the NMRI reference genome (1.35 and 1.96 mutated site per kb), which was clearly higher than the values (*i.e.* between 0.312 and 0.857 per kb) found after the re-sequencing of lab strains from *Entamoeba histolytica*, another human parasite [56] ; (ii) a large majority of SNPs (90.8%) we identified were private, either to BRE (35.8%) or GH2 (55.0%) strains, indicating specific polymorphic loci; (iii) mean fixation index was clearly very high, certainly as a result of the only few shared and variable SNPs (Table 1) and (iv) large differences in copy number variations were identified between both strains. All these genome-wide diversity results are fully consistent with the geographic separation of the two strains in the wild since their introduction in the New World from Africa, hampering any genetic exchange, and separate cultivation for decades in the laboratory (Supplementary figure S1). Interestingly, rare SNPs represent the most important class of private SNPs, thus demonstrating that long maintenance in the lab conditions, even if it would largely decrease diversity, could not fully erase segregating polymorphisms.

Using SNP frequencies within each population, we applied a recently published method of population genomics to identify selective sweeps [21], [22]. The sweep detection method of Pool-hmm [21], [22] uses both the density of segregating sites and the allele frequency pattern among segregating sites to distinguish sweep regions from neutral regions. In populations with low genetic diversity the length of regions with low genetic diversity in the genome is expected to be higher and therefore mapping resolution lower. Low genetic diversity of our populations will affect the power of the method (false negative sweep detection rate) and the resolution of the sweeps but not its robustness (false positive sweep detection rate). The high number of selective sweeps for both BRE and GH2 strains indicated that a high selective pressure has operated at the whole genome scale. Attributing these signatures of selection to either long evolutionary history or adaptation to laboratory conditions will now require comparison with strains sampled from the field. We therefore focused on private (*i.e.* specific non-overlapping) selective sweeps that may not correspond to adaptation to the common laboratory conditions but to long-term evolutionary processes instead. It also helped to increase resolution because the private selective sweeps all constituted smaller regions than other sweeps. We may have here under-estimated the number of regions involved in adaptation and missed some relevant regions that would be identified in further work on field strains. The robustness of the method we used ensures a true and confident discovery of private sweeps, which was confirmed by the high proportion of non-synonymous SNPs that was found within selective sweeps (Supplementary figure S6).

As differences in copy number variation (CNV) within a same species have been recognized to be involved in adaptive evolution [25], [26], [57], we also investigated such variations as clues of selection between BRE and GH2. We used the recently published CNV-seq method [24] that takes advantage of the high throughput sequencing data and is suitable for pairwise comparisons. The high number (2,003 in total) of CNV found across the whole-genome of *S. mansoni* reinforced our hypothesis of high selective pressure and even suggested that these selective pressures were different between both strains and contributed to shape the specific genomic landscape observed for each strain.

Functional annotations and analyses gave more biological significance to these specific signatures of selection. Exploring private selective sweeps, we identified 592 (BRE) and 791 (GH2) transcripts that have been potentially subjected to selection, but some of them could also have been identified because of their genomic proximity with truly selected genes. We therefore regrouped genes in regions under selection and/or with CNV by function using GO terms. Genes that did not form functional groups (outliers) were considered as having little or no significance. Overall, biological processes specific to each strain emerged from functional analysis, among which there is cell-cell adhesion for BRE and reduction-oxidation reactions, potentially involved in ROS production or ROS scavenging, for GH2. Earlier studies postulated that an evolutionary arms race between snail host and parasite operates preferentially on immune effectors for the Brazilian strain and on immune recognition for the Guadeloupean strain [3], [42]. This is consistent with negative selection process acting on these pathways in the two strains. Two pathways involved in N-glycan biosynthesis were also found under selection in BRE. This finding was consistent with previously observed differences between BRE and GH2 in their glycosylation level for SmPoMucs [51]. As SmPoMucs were shown to be involved in compatibility polymorphism, and because the level of glycosylation is directly related to compatibility, we argue that differential selection in the N-glycan biosynthesis pathway may be responsible for this compatibility polymorphism between the two strains. In summary, we find correspondence between observed life-history traits of the different strains and genomic regions under selection. However, we see this rather on the biological function and metabolic pathway level and not on the level of individual genes.

Despite strong genomic divergence between BRE and GH2 strains, functional analyses revealed interesting evolutionary convergence. From non-overlapping lists of transcripts (because only private sweeps were analysed), we highlighted two common biological processes and three common molecular pathways as targets of selection. More particularly, we independently identified numerous proteases in genomic swept regions or in regions showing copy number variations in both strains, indicating an evolutionary convergence to this function. Proteases are indeed key virulence factors of parasites and particularly trematodes, as they are involved in a number of biological processes such as host tissue invasion/migration, nutrition from host substrates (*e.g.* haemoglobin degradation), immune evasion and more generally host-parasite interactions [58], [59]. Phylogenomic analysis had shown that protease-encoding genes, including cathepsins for which we found two genes under selection in the BRE strain, were expanded in the *Schistosoma* lineage [60]. Cathepsins are secreted proteases and due to their importance in host-parasite interactions they are considered to be promising targets for the development of novel chemotherapeutic drugs and vaccines against schistosomiasis. Simoes *et al.* [61] previously described SNPs in the cysteine protease Cathepsin B vaccine target gene that are involved in significant conformation changes leading to an alteration in antibody binding to the protein. Variability among *Schistosoma* species at the biochemical level has been described earlier for cathepsin B-like activity [62]. Our results suggest that different selective pressures operated on this gene, and we propose that using this as a target for vaccine development could rapidly become inefficient in natural populations. Cathepsin D is an aspartyl protease originating from successive gene duplication events in the parasite lineage after its diversification from other metazoans and was proposed to be involved in adaptation to the parasitic lifestyle [49]. Here, we also showed that this gene could be under differential selection between strains of the *S. mansoni* species. Further analyses of these two particular genes have to be conducted to investigate the functional basis of this evolutionary signature. From CNV data, we identified an M13 family peptidase, a type II membrane metallo-endopeptidases acting on extracellular substrates [48], belonging to a family of proteases with other member involved in one of the epigenetic differences found between these two strains in a previous study [12]. Characterized members of the family such as neprilysin act on polypeptides smaller than 40 amino-acids. Considering evolutionary relationships among members of the family, it has been proposed that proteases of this family fulfil a broad range of physiological roles [63]. In nematodes, a neprilysin-like protease is involved in locomotion and pharyngeal activity [64]. Combination of our results indicated that the differences in BRE and GH2 life history traits [3], [11] could result from the selection and maintenance of different proteases, leading for example to different compatibility levels with the intermediate host. Altogether, these elements support the hypothesis that the proteolysis function has evolved through different ways within different populations of the *S. mansoni* species. Evolutionary convergence was generally described between species [65] or communities [66] to explain adaptation of different species to a particular life style. Proteolysis could thus be a promising function to further focus on because of its key role in parasitic lifestyle, either to better understand parasite evolution and adaptation to its hosts or to develop new treatment or preventive strategies. Since the function of most genes in the *S. mansoni* genome has not yet been confirmed by functional studies, further investigations are nevertheless needed to objectively demonstrate the above conclusions.

It is important to note that the 47% of repetitive elements in the *S. mansoni* genome were not investigated in this work because we excluded all reads that matched more than one time to the genome to allow SNP and CNV identification. We may have thus under-estimated the number of regions under selection. The recent *de novo* repeat assembly [31] should facilitate such a study on the repetitive part of the genome. The identification of SNPs in segmental duplications could also open new perspectives in *S. mansoni* evolutionary studies. As gene duplication is a mechanism of genomic adaptation to a changing environment [67], it could be largely involved in parasite adaptation as it was suggested earlier with peptidase families [49].

In conclusion, our work provides one of the first examples of a comparative genomic approach based on population sequencing. A genome-wide comparison of single-nucleotide and structural polymorphisms combined with population and functional analyses allowed us here to identify signatures of selection in two *S. mansoni* populations. Even if all the life history traits and proteome complexity have not found their genetic bases through this study, we were able to identify selection acting on specific functions involved in parasitic lifestyle. Notably, our integrative approach highlighted that selection can act on a same function but through different pools of genes, which clearly suggests evolutionary convergence within schistosomes. However, high-throughput genotyping of a larger number of populations (with the pool-sequencing method) is expected to shed more light into evolutionary history and bases of adaptation of *Schistosoma mansoni*. In particular further comparative analyses of field isolates of Old World and New World strains could help to better understand the evolutionary history and diversification of the parasite since its “out-of-Africa” origin [68]. Such future studies would also help us to identify more polymorphisms associated with the intermediate host compatibility and to clarify how the rapid adaptation of the parasite to *Biomphalaria glabrata* has occurred during the colonization of the New World.

## Supporting Information

Figure S1Schematic representation of the history of the two strains used in this study and of the relation between phenotypic and genetic parameters along the chronology of events. On the left (“History”) the known isolation and bottleneck events 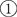 divergence of the two original wild populations from the African origin. 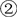 sampling of individuals leading to the lab-populations for two respective populations 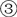 maintenance of the two populations in the laboratory for thirty years, in the middle (“Life history traits”) representation of the shifts in life history traits for both strains, Bre in grey, GH2 in black. The during the out of Africa migration is hypothetical, but we know that phenotypic characters remained stable during 30 years in the laboratory. On the right (“Genetic diversity”), hypothetical shifts in genetic diversity that could have led to the observed FST and Θ.(PDF)

Figure S2 Repartition of SNPs number (bars) and density (dots) between chromosomes for the Brazilian (A) and the Guadeloupean (B) strains of *Schistosoma mansoni* used in this study.
(PDF)

Figure S3 Distribution of SNP number in 10 kb-window across each of the seven autosomes (A to G) and across the ZW-linkage group (H) of *Schistosoma mansoni*.
(PDF)

Figure S4 Log 2 ratio plot of copy number variations for the largest CNV genomic region.
(PDF)

Figure S5 Distribution of cellular processes found under selection for the Brazilian (A) and Guadeloupean (B) strains based on their Gene Ontology term at the level 2.
(PDF)

Figure S6 Non-synonymous (NonSyn-) and synonymous (Syn-) SNPs distribution within and outside selective sweeps found in the genome of *Schistosoma mansoni*. Numbers represent the amount of SNPs in each proportion, with the details for specific non-overlapping regions we focused on in this study.
(PDF)

Table S1 Number and proportion of reads conserved after each step of bioinformatic treatment of Brazilian (BRE) and Guadeloupean (GH2) strains of *Schistosoma mansoni*.
(DOCX)

Table S2 Excel file with summary of nucleotide diversity measures of Tajima's D and Watterson' Theta for the 7 autosomes of BRE and GH2 strains. See excel file Table_S2_SummaryResPopoolation.xls.
(XLS)

Table S3 Excel file with list of genomic regions with copy number variation between BRE and GH2. See excel file Table_S3_CNV_list.xlsx.
(XLS)

Table S4 Protein-coding genes in the Top 20 CNV regions (by length) in *Schistosoma mansoni* BRE and GH2.
(DOCX)

Table S5 Protein-coding genes in the Top 20 CNV regions (by log2ratio) in *Schistosoma mansoni* strains BRE and GH2.
(DOCX)

Table S6 List of transcripts identified in private selective sweeps for BRE (A) and GH2 (B) strains. See excel file Table_S6_transcripts_in_private_selective_sweeps.xls.
(XLS)

Table S7 Common and specific pathways in which enzymes encoded by genes in genomic regions under selection were found for BRE and GH2 strains.
(DOCX)

Table S8 Primers used for the polymerase chain reaction (PCR) to check *in situ* 22 single nucleotide polymorphisms of both Brazilian and Guadeloupean strains of *Schistosoma mansoni* used in this study.
(DOCX)

Table S9 Number of selective sweeps detected on autosomes of *Schistosoma mansoni* strains BRE and GH2 used in this study for two values of transition probability *q*.
(DOCX)

## References

[1] Carter NP (2007) Methods and strategies for analyzing copy number variation using DNA microarrays. Nat Genet 39: S16–S21 doi:10.1038/ng2028 17597776 10.1038/ng2028 PMC2697494

[2] Ridley M (2009) Evolution. Wiley-Blackwell. 1 pp.

[3] Roger E, Mitta G, Mone Y, Bouchut A, Rognon A, et al (2008) Molecular determinants of compatibility polymorphism in the Biomphalaria glabrata/Schistosoma mansoni model: New candidates identified by a global comparative proteomics approach. Mol Biochem Parasitol 157: 205–216 doi:10.1016/j.molbiopara.2007.11.003 18083248 10.1016/j.molbiopara.2007.11.003

[4] Theron A, Coustau C (2005) Are Biomphalaria snails resistant to Schistosoma mansoni? J Helminthol 79: 187–191 doi:10.1079/joh2005299 16153311 10.1079/joh2005299

[5] Webster JP, Davies CM (2001) Coevolution and compatibility in the snail-schistosome system. Parasitology 123: S41–S56. 11769292 10.1017/s0031182001008071

[6] El-Ansary A, Al-Daihan S (2006) Important aspects ofBiomphalariasnail-schistosome interactions as targets for antischistosome drug. Medical Science Monitor 12: RA282–RA292. 17136018

[7] Theron A, Pages JR, Rognon A (1997) Schistosoma mansoni: Distribution patterns of miracidia among Biomphalaria glabrata snail as related to host susceptibility and sporocyst regulatory processes. Exp Parasitol 85: 1–9 doi:10.1006/expr.1996.4106 9024196 10.1006/expr.1996.4106

[8] Chitsulo L, Engels D, Montresor A, Savioli L (2000) The global status of schistosomiasis and its control. Acta Trop 77: 41–51 doi:10.1016/s0001-706x(00)00122-4 10996119 10.1016/s0001-706x(00)00122-4 PMC5633072

[9] Chitsulo L, Loverde R, Engels D, Barakat R, Colley D, et al (2004) Schistosomiasis. Nat Rev Micro 2: 12–13 doi:10.1038/nrmicro801 10.1038/nrmicro801 15035004

[10] King CH (2010) Parasites and poverty: The case of schistosomiasis. Acta Trop 113: 95–104 doi:10.1016/j.actatropica.2009.11.012 19962954 10.1016/j.actatropica.2009.11.012 PMC2812649

[11] Theron A (1980) Mise en évidence de races chonobiologiques de Schistosoma mansoni, agent de la bilharziose, à partir de cinétiques d'émission cercarienne. Comptes Rendus de l'Académie des Sciences, Paris. 4 pp.

[12] Lepesant JMJ, Grunau C, Cosseau C (2011) Towards an understanding of the epigenetics of schistosomes: a comparative epigenomic study. Mem Inst Oswaldo Cruz 106: 823–830. 22124554 10.1590/s0074-02762011000700007

[13] Tajima F (1989) Statistical method for testing the neutral mutation hypothesis by DNA polymorphism. Genetics 123: 585–595. 2513255 10.1093/genetics/123.3.585 PMC1203831

[14] Suzuki Y (2010) Statistical methods for detecting natural selection from genomic data. Genes Genet Syst 85: 359–376 doi:10.1266/ggs.85.359 21415566 10.1266/ggs.85.359

[15] Nielsen R (2005) Molecular signatures of natural selection. Annual Review of Genetics, Vol 44. Palo Alto: Annual Reviews, Vol. 39. pp. 197–218. doi:10.1146/annurev.genet.39.073003.112420. 10.1146/annurev.genet.39.073003.112420 16285858

[16] Zayed A, Whitfield CW (2008) A genome-wide signature of positive selection in ancient and recent invasive expansions of the honey bee Apis mellifera. Proc Natl Acad Sci USA 105: 3421–3426 doi:10.1073/pnas.0800107105 18299560 10.1073/pnas.0800107105 PMC2265178

[17] Akey JM (2002) Interrogating a High-Density SNP Map for Signatures of Natural Selection. Genome Research 12: 1805–1814 doi:10.1101/gr.631202 12466284 10.1101/gr.631202 PMC187574

[18] Biswas S, Akey JM (2006) Genomic insights into positive selection. Trends Genet 22: 437–446 doi:10.1016/j.tig.2006.06.005 16808986 10.1016/j.tig.2006.06.005

[19] Gautier M, Flori L, Riebler A, Jaffrezic F, Laloe D, et al (2009) A whole genome Bayesian scan for adaptive genetic divergence in West African cattle. BMC Genomics 10: 550 doi:10.1186/1471-2164-10-550 19930592 10.1186/1471-2164-10-550 PMC2784811

[20] Boitard S, Schlötterer C, Futschik A (2009) Detecting selective sweeps: a new approach based on hidden markov models. Genetics 181: 1567–1578 doi:10.1534/genetics.108.100032 19204373 10.1534/genetics.108.100032 PMC2666521

[21] Boitard S, Schlotterer C, Nolte V, Pandey RV, Futschik A (2012) Detecting Selective Sweeps from Pooled Next-Generation Sequencing Samples. Molecular Biology and Evolution 29: 2177–2186 doi:10.1093/molbev/mss090 22411855 10.1093/molbev/mss090 PMC3424412

[22] Boitard S, Kofler R, Françoise P, Robelin D, Schlötterer C, et al (2013) Pool-hmm: a Python program for estimating the allele frequency spectrum and detecting selective sweeps from next generation sequencing of pooled samples. Mol Ecol Resour 13: 337–340 doi:10.1111/1755-0998.12063 23311589 10.1111/1755-0998.12063 PMC3592992

[23] Gautier M, Vitalis R (2013) Inferring population histories using genome-wide allele frequency data. Molecular Biology and Evolution 30: 654–668 doi:10.1093/molbev/mss257 23155004 10.1093/molbev/mss257

[24] Xie C, Tammi MT (2009) CNV-seq, a new method to detect copy number variation using high-throughput sequencing. BMC Bioinformatics 10: 80 doi:10.1186/1471-2105-10-80 19267900 10.1186/1471-2105-10-80 PMC2667514

[25] Lynch M (2002) Genomics - Gene duplication and evolution. Science 297: 945–947 doi:10.1126/science.1075472 12169715 10.1126/science.1075472

[26] Lynch M, Conery JS (2000) The evolutionary fate and consequences of duplicate genes. Science 290: 1151–1155 doi:10.1126/science.290.5494.1151 11073452 10.1126/science.290.5494.1151

[27] Protasio AV, Tsai IJ, Babbage A, Nichol S, Hunt M, et al (2012) A Systematically Improved High Quality Genome and Transcriptome of the Human Blood Fluke Schistosoma mansoni. PLoS Negl Trop Dis 6: e1455 doi:10.1371/journal.pntd.0001455 22253936 10.1371/journal.pntd.0001455 PMC3254664

[28] Bech N, Beltran S, Portela J, Rognon A, Allienne J-F, et al (2010) Follow-up of the genetic diversity and snail infectivity of a Schistosoma mansoni strain from field to laboratory. Infection, Genetics and Evolution 10: 1039–1045 doi:10.1016/j.meegid.2010.06.012 10.1016/j.meegid.2010.06.012 20601175

[29] Maniatis T, Sambrook J, Fritsch EF (1982) Molecular Cloning - A laboratory manual. Cold Spring Harbor, New York: Cold Spring Harbor Press.

[30] Langmead B, Trapnell C, Pop M, Salzberg SL (2009) Ultrafast and memory-efficient alignment of short DNA sequences to the human genome. Genome Biol 10: R25 doi:10.1186/gb-2009-10-3-r25 19261174 10.1186/gb-2009-10-3-r25 PMC2690996

[31] Lepesant JMJ, Roquis D, Emans R, Cosseau C, Arancibia N, et al (2012) Combination of de novo assembly of massive sequencing reads with classical repeat prediction improves identification of repetitive sequences in Schistosoma mansoni. Exp Parasitol 130: 470–474 doi:10.1016/j.exppara.2012.02.010 22381218 10.1016/j.exppara.2012.02.010

[32] Marth G, Yeh R, Minton M, Donaldson R, Li Q, et al (2001) Single-nucleotide polymorphisms in the public domain: how useful are they? Nat Genet 27: 371–372 doi:10.1038/86864 11279516 10.1038/86864

[33] Li H, Handsaker B, Wysoker A, Fennell T, Ruan J, et al (2009) The Sequence Alignment/Map format and SAMtools. Bioinformatics 25: 2078–2079 doi:10.1093/bioinformatics/btp352 19505943 10.1093/bioinformatics/btp352 PMC2723002

[34] Giardine B, Riemer C, Hardison RC, Burhans R, Elnitski L, et al (2005) Galaxy: A platform for interactive large-scale genome analysis. Genome Research 15: 1451–1455 doi:10.1101/gr.4086505 16169926 10.1101/gr.4086505 PMC1240089

[35] Futschik A, Schlötterer C (2010) The next generation of molecular markers from massively parallel sequencing of pooled DNA samples. Genetics 186: 207–218 doi:10.1534/genetics.110.114397 20457880 10.1534/genetics.110.114397 PMC2940288

[36] Kofler R, Orozco-terwengel P, De Maio N, Pandey RV, Nolte V, et al (2011) PoPoolation: a toolbox for population genetic analysis of next generation sequencing data from pooled individuals. PLoS ONE 6: e15925 doi:10.1371/journal.pone.0015925 21253599 10.1371/journal.pone.0015925 PMC3017084

[37] Kofler R, Pandey RV, Schlotterer C (2011) PoPoolation2: identifying differentiation between populations using sequencing of pooled DNA samples (Pool-Seq). Bioinformatics 27: 3435–3436 doi:10.1093/bioinformatics/btr589 22025480 10.1093/bioinformatics/btr589 PMC3232374

[38] Conesa A, Gotz S, Garcia-Gomez JM, Terol J, Talon M, et al (2005) Blast2GO: a universal tool for annotation, visualization and analysis in functional genomics research. Bioinformatics 21: 3674–3676 doi:10.1093/bioinformatics/bti610 16081474 10.1093/bioinformatics/bti610

[39] Zdobnov EM, Apweiler R (2001) InterProScan - an integration platform for the signature-recognition methods in InterPro. Bioinformatics 17: 847–848 doi:10.1093/bioinformatics/17.9.847 11590104 10.1093/bioinformatics/17.9.847

[40] R Development Core Team (2005) R: a language and environment for statistical computing.

[41] Roger E, Gourbal B, Grunau C, Pierce RJ, Galinier R, et al (2008) Expression analysis of highly polymorphic mucin proteins (Sm PoMuc) from the parasite Schistosoma mansoni. Mol Biochem Parasitol 157: 217–227 doi:10.1016/j.molbiopara.2007.11.015 18187213 10.1016/j.molbiopara.2007.11.015

[42] Mitta G, Adema CM, Gourbal B, Loker ES, Theron A (2012) Compatibility polymorphism in snail/schistosome interactions: From field to theory to molecular mechanisms. Dev Comp Immunol 37: 1–8 doi:10.1016/j.dci.2011.09.002 21945832 10.1016/j.dci.2011.09.002 PMC3645982

[43] Amos W (2010) Even small SNP clusters are non-randomly distributed: is this evidence of mutational non-independence? Proc R Soc B-Biol Sci 277: 1443–1449 doi:10.1098/rspb.2009.1757 10.1098/rspb.2009.1757 PMC2871933 20071383

[44] Lindblad-Toh K, Winchester E, Daly MJ, Wang DG, Hirschhorn JN, et al (2000) Large-scale discovery and genotyping of single-nucleotide polymorphisms in the mouse. Nat Genet 24: 381–386 doi:10.1038/74215 10742102 10.1038/74215

[45] Drake JW, Charlesworth B, Charlesworth D, Crow JF (1998) Rates of spontaneous mutation. Genetics 148: 1667–1686. 9560386 10.1093/genetics/148.4.1667 PMC1460098

[46] Lynch M (2010) Evolution of the mutation rate. Trends Genet 26: 345–352 doi:10.1016/j.tig.2010.05.003 20594608 10.1016/j.tig.2010.05.003 PMC2910838

[47] Bradshaw AD (2012) Diverse biological functions of the SPARC family of proteins. Int J Biochem Cell Biol 44: 480–488 doi:10.1016/j.biocel.2011.12.021 22249026 10.1016/j.biocel.2011.12.021 PMC3312742

[48] Oefner C, D'Arcy A, Hennig M, Winkler FK, Dale GE (2000) Structure of human neutral endopeptidase (Neprilysin) complexed with phosphoramidon. J Mol Biol 296: 341–349 doi:10.1006/jmbi.1999.3492 10669592 10.1006/jmbi.1999.3492

[49] Silva LL, Marcet-Houben M, Zerlotini A, Gabaldon T, Oliveira G, et al (2011) Evolutionary histories of expanded peptidase families in Schistosoma mansoni. Mem Inst Oswaldo Cruz 106: 864–877. 22124560 10.1590/s0074-02762011000700013

[50] Sajid M, McKerrow JH, Hansell E, Mathieu MA, Lucas KD, et al (2003) Functional expression and characterization ofSchistosoma mansonicathepsin B and its trans-activation by an endogenous asparaginyl endopeptidase. Mol Biochem Parasitol 131: 65–75 doi:10.1016/s0166-6851(03)00194-4 12967713 10.1016/s0166-6851(03)00194-4

[51] Roger E, Grunau C, Pierce RJ, Hirai H, Gourbal B, et al (2008) Controlled Chaos of Polymorphic Mucins in a Metazoan Parasite (Schistosoma mansoni) Interacting with Its Invertebrate Host (Biomphalaria glabrata). PLoS Negl Trop Dis 2: e330 doi:10.1371/journal.pntd.0000330 19002242 10.1371/journal.pntd.0000330 PMC2576457

[52] Berriman M, Haas BJ, Loverde PT, Wilson RA, Dillon GP, et al (2009) The genome of the blood fluke Schistosoma mansoni. Nature 460: 352–358 doi:10.1038/nature08160 19606141 10.1038/nature08160 PMC2756445

[53] Cosseau C, Azzi A, Rognon A, Boissier J, Gourbiere S, et al (2010) Epigenetic and phenotypic variability in populations of Schistosoma mansoni - a possible kick-off for adaptive host/parasite evolution. Oikos 119: 669–678 doi:10.1111/j.1600-0706.2009.18040.x

[54] Branca A, Paape TD, Zhou P, Briskine R, Farmer AD, et al (2011) Whole-genome nucleotide diversity, recombination, and linkage disequilibrium in the model legume Medicago truncatula. Proceedings of the National Academy of Sciences 108: E864–E870 doi:10.1073/pnas.1104032108 10.1073/pnas.1104032108 PMC3198318 21949378

[55] Yanagida T, Mohammadzadeh T, Kamhawi S, Nakao M, Sadjjadi SM, et al (2012) Genetic polymorphisms of Echinococcus granulosus sensu stricto in the Middle East. Parasitol Int 61: 599–603 doi:10.1016/j.parint.2012.05.014 22668837 10.1016/j.parint.2012.05.014

[56] Weedall GD, Clark CG, Koldkjaer P, Kay S, Bruchhaus I, et al (2012) Genomic diversity of the human intestinal parasite Entamoeba histolytica. Genome Biol 13: R38 doi:10.1186/gb-2012-13-5-r38 22630046 10.1186/gb-2012-13-5-r38 PMC3446291

[57] Gemayel R, Vinces MD, Legendre M, Verstrepen KJ (2010) Variable Tandem Repeats Accelerate Evolution of Coding and Regulatory Sequences. Annu Rev Genet 44: 445–477 doi:10.1146/annurev-genet-072610-155046 20809801 10.1146/annurev-genet-072610-155046

[58] McKerrow JH, Caffrey C, Kelly B, Loke P, Sajid M (2006) Proteases in parasitic diseases. Annual Review of Pathology-Mechanisms of Disease. Palo Alto: Annual Reviews, Vol. 1. pp. 497–536. doi:10.1146/annurev.pathol.1.110304.100151. 10.1146/annurev.pathol.1.110304.100151 18039124

[59] Kasny M, Mikes L, Hampl V, Dvorak J, Caffrey CR, et al.. (2009) Peptidases of Trematodes. In: Rollinson D, Hay SI, editors. Advances in Parasitology. Vol. 69. pp. 205—. doi:10.1016/s0065-308x(09)69004-7. 10.1016/S0065-308X(09)69004-7 19622410

[60] Silva LL, Marcet-Houben M, Nahum LA, Zerlotini A, Gabaldón T, et al (2012) The Schistosoma mansoni phylome: using evolutionary genomics to gain insight into a parasite's biology. BMC Genomics 13: 617 doi:10.1186/1471-2164-13-617 23148687 10.1186/1471-2164-13-617 PMC3534613

[61] Simoes M, Bahia D, Zerlotini A, Torres K, Artiguenave F, et al (2007) Single nucleotide polymorphisms identification in expressed genes of Schistosoma mansoni. Mol Biochem Parasitol 154: 134–140 doi:10.1016/j.moibiopara.2007.04.003 17568698 10.1016/j.molbiopara.2007.04.003 PMC1986741

[62] Caffrey CR, Rheinberg CE, Moné H, Jourdane J, Li YL, et al (1997) Schistosoma japonicum, S. mansoni, S. haematobium, S. intercalatum, and S. rodhaini: cysteine-class cathepsin activities in the vomitus of adult worms. Parasitol Res 83: 37–41. 9000231 10.1007/s004360050204

[63] Bland ND, Pinney JW, Thomas JE, Turner AJ, Isaac RE (2008) Bioinformatic analysis of the neprilysin (M13) family of peptidases reveals complex evolutionary and functional relationships. BMC Evol Biol 8: 16 doi:10.1186/1471-2148-8-16 18215274 10.1186/1471-2148-8-16 PMC2259306

[64] Spanier B, Stürzenbaum SR, Holden-Dye LM, Baumeister R (2005) Caenorhabditis elegans neprilysin NEP-1: an effector of locomotion and pharyngeal pumping. J Mol Biol 352: 429–437 doi:10.1016/j.jmb.2005.06.063 16081104 10.1016/j.jmb.2005.06.063

[65] Srithayakumar V, Castillo S, Mainguy J, Kyle CJ (2012) Evidence for evolutionary convergence at MHC in two broadly distributed mesocarnivores. Immunogenetics 64: 289–301 doi:10.1007/s00251-011-0588-7 22085968 10.1007/s00251-011-0588-7

[66] Fan L, Reynolds D, Liu M, Stark M, Kjelleberg S, et al (2012) Functional equivalence and evolutionary convergence in complex communities of microbial sponge symbionts. Proceedings of the National Academy of Sciences 109: E1878–E1887 doi:10.1073/pnas.1203287109 10.1073/pnas.1203287109 PMC3390844 22699508

[67] Kondrashov FA (2012) Gene duplication as a mechanism of genomic adaptation to a changing environment. Proc R Soc B-Biol Sci 279: 5048–5057 doi:10.1098/rspb.2012.1108 10.1098/rspb.2012.1108 PMC3497230 22977152

[68] Morgan JAT, Dejong RJ, Adeoye GO, Ansa EDO, Barbosa CS, et al (2005) Origin and diversification of the human parasite Schistosoma mansoni. Molecular Ecology 14: 3889–3902 doi:10.1111/j.1365-294X.2005.02709.x 16202103 10.1111/j.1365-294X.2005.02709.x

