# Supplementary figures and images for: Private Selective Sweeps Identified from Next-Generation Pool-Sequencing Reveal Convergent Pathways under Selection in Two Inbred Schistosoma mansoni Strains
Source: PLoS Negl Trop Dis. 2013 Dec 12;7(12):e2591. doi: 10.1371/journal.pntd.0002591 (PMC3861164; doi:10.1371/journal.pntd.0002591)

Figure S1

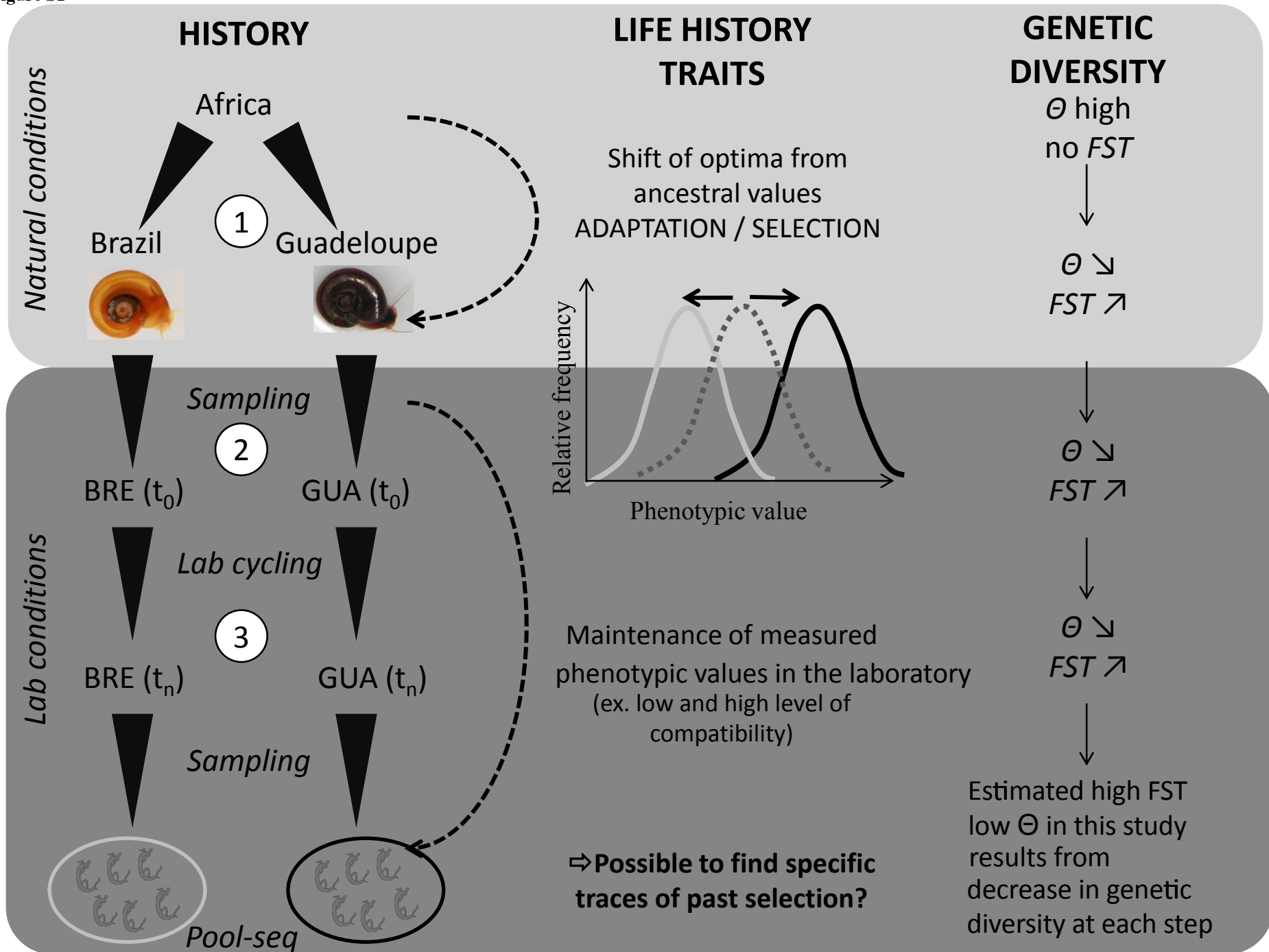

Supplement: Figure S1 — Schematic representation of the history of the two strains used in this study and of the relation between phenotypic and genetic parameters along the chronology of events. On the left (“History”) the known isolation and bottleneck events divergence of the two original wild populations from the African origin. sampling of individuals leading to the lab-populations for two respective populations maintenance of the two populations in the laboratory for thirty years, in the middle (“Life history traits”) representation of the shifts in life history traits for both strains, Bre in grey, GH2 in black. The during the out of Africa migration is hypothetical, but we know that phenotypic characters remained stable during 30 years in the laboratory. On the right (“Genetic diversity”), hypothetical shifts in genetic diversity that could have led to the observed FST and Θ. (PDF) [file pntd.0002591.s001.pdf]

**Figure S2**

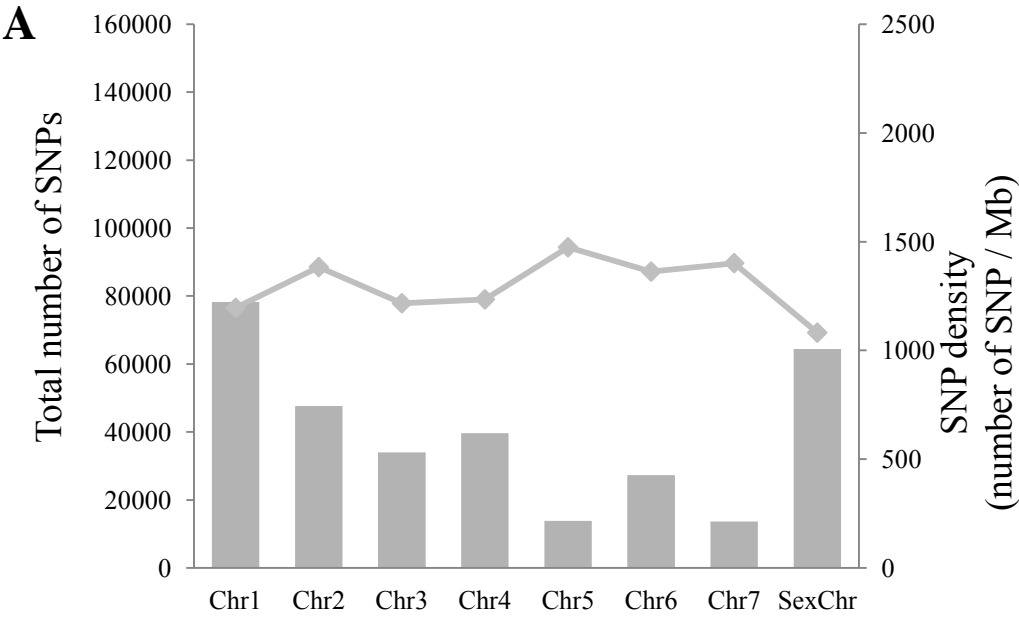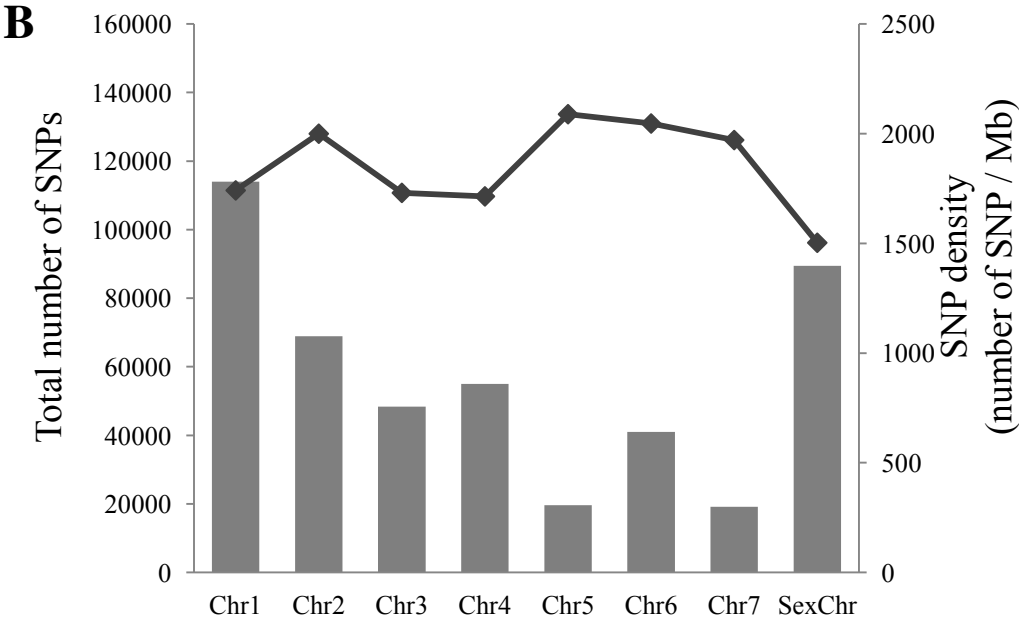

Supplement: Figure S2 — Repartition of SNPs number (bars) and density (dots) between chromosomes for the Brazilian (A) and the Guadeloupean (B) strains of Schistosoma mansoni used in this study. (PDF) [file pntd.0002591.s002.pdf]

**Figure S4**

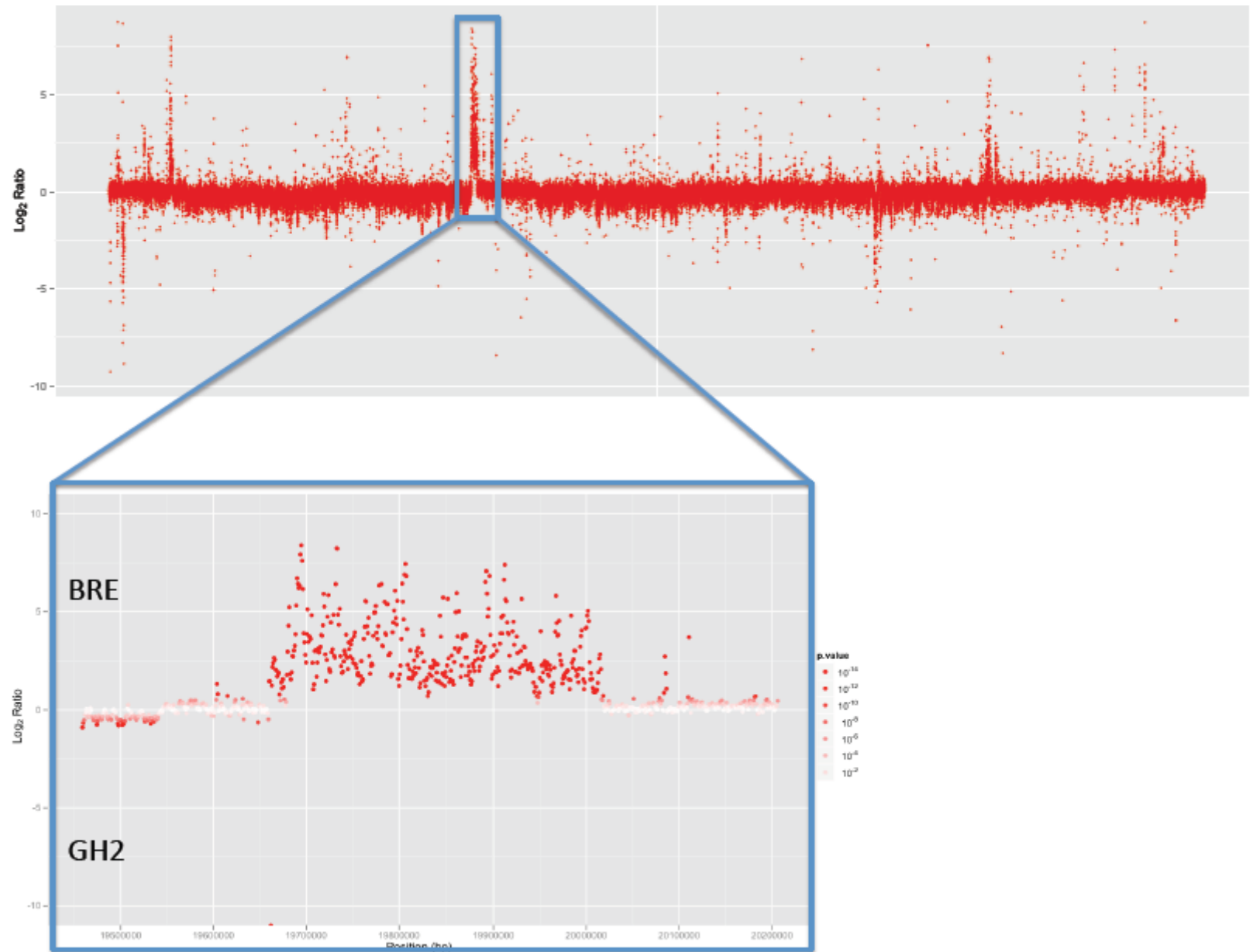

Supplement: Figure S4 — Log 2 ratio plot of copy number variations for the largest CNV genomic region. (PDF) [file pntd.0002591.s004.pdf]

**Figure S5**

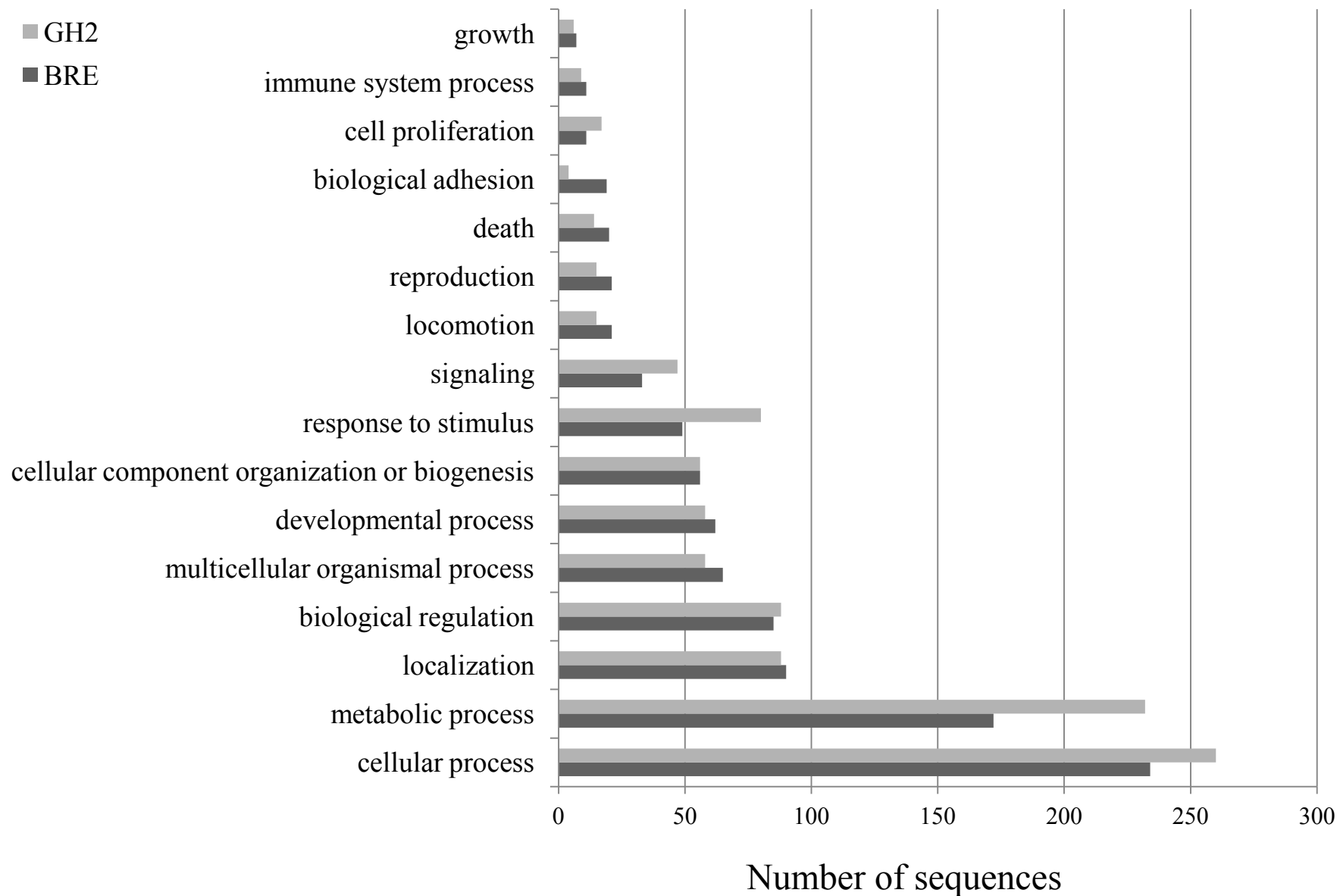

Supplement: Figure S5 — Distribution of cellular processes found under selection for the Brazilian (A) and Guadeloupean (B) strains based on their Gene Ontology term at the level 2. (PDF) [file pntd.0002591.s005.pdf]

**Figure S6**

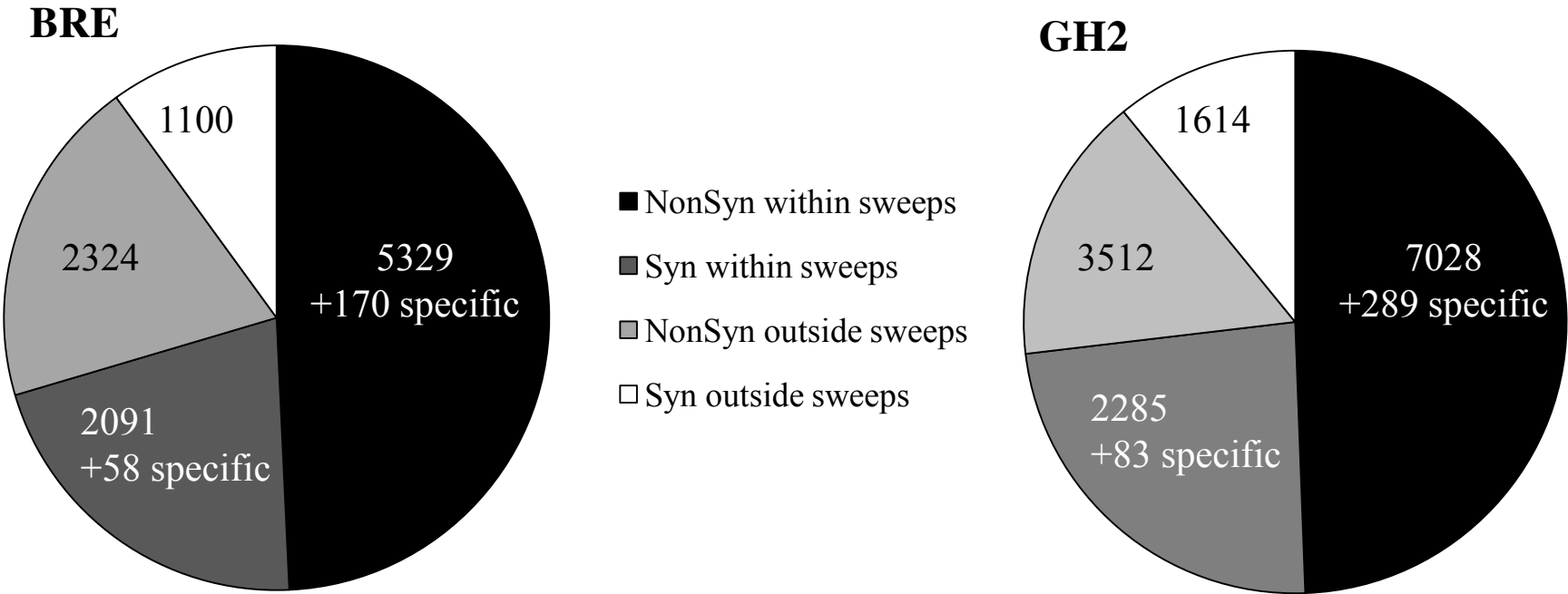

Supplement: Figure S6 — Non-synonymous (NonSyn-) and synonymous (Syn-) SNPs distribution within and outside selective sweeps found in the genome of Schistosoma mansoni. Numbers represent the amount of SNPs in each proportion, with the details for specific non-overlapping regions we focused on in this study. (PDF) [file pntd.0002591.s006.pdf]
