# Supplementary material for: Private Selective Sweeps Identified from Next-Generation Pool-Sequencing Reveal Convergent Pathways under Selection in Two Inbred Schistosoma mansoni Strains
Source: PLoS Negl Trop Dis. 2013 Dec 12;7(12):e2591. doi: 10.1371/journal.pntd.0002591 (PMC3861164; doi:10.1371/journal.pntd.0002591)

Figure S3A

CHROMOSOME 1

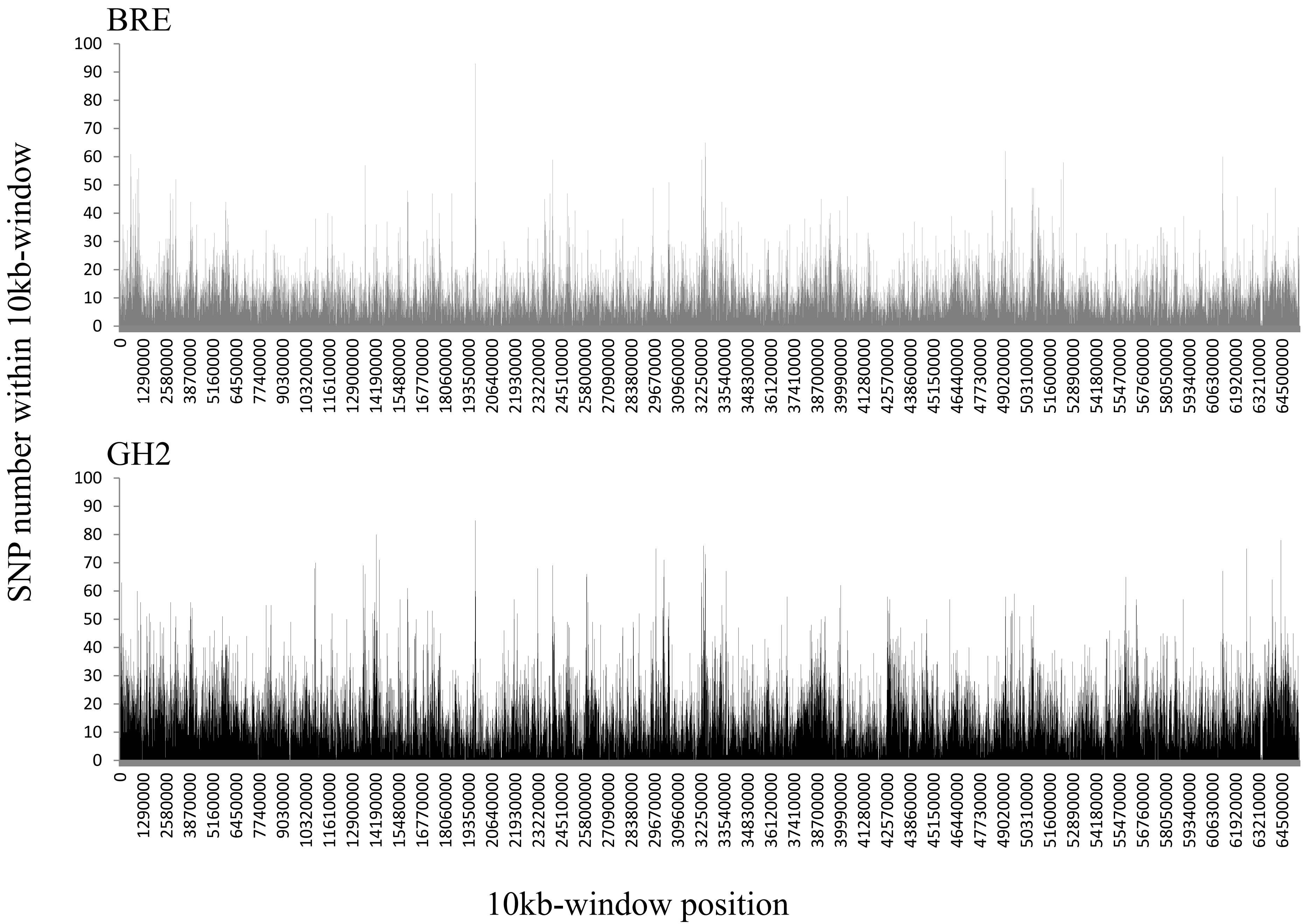

Figure S3 B

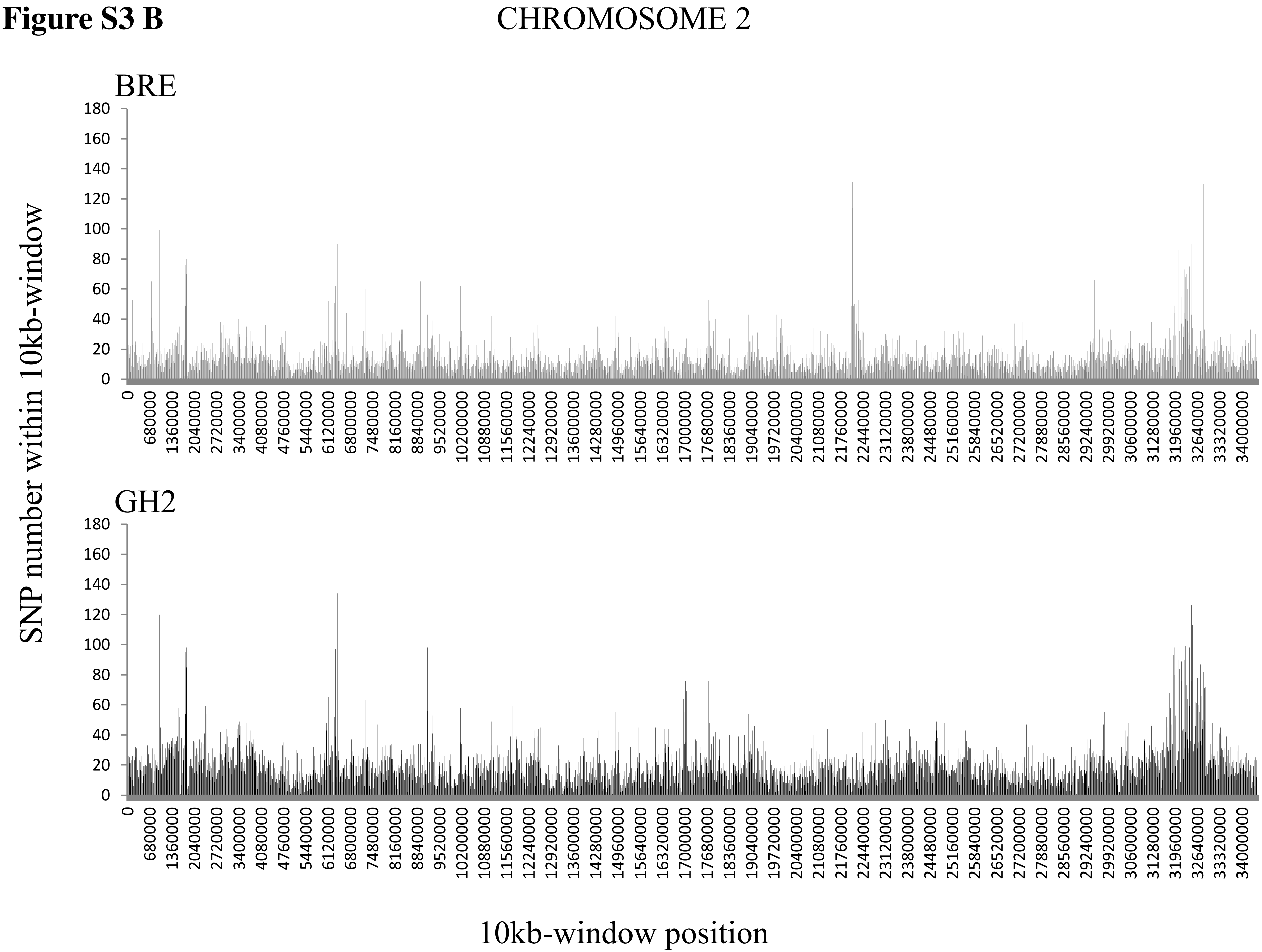
CHROMOSOME 2
BRE
GH2
SNP number within 10kb-window
10kb-window position
0
20
40
60
80
100
120
140
160
180
0
680000
1360000
2040000
2720000
3400000
4080000
4760000
5440000
6120000
6800000
7480000
8160000
8840000
9520000
10200000
10880000
11560000
12240000
12920000
13600000
14280000
14960000
15640000
16320000
17000000
17680000
18360000
19040000
19720000
20400000
21080000
21760000
22440000
23120000
23800000
24480000
25160000
25840000
26520000
27200000
27880000
28560000
29240000
29920000
30600000
31280000
31960000
32640000
33320000
34000000

Figure S3 C

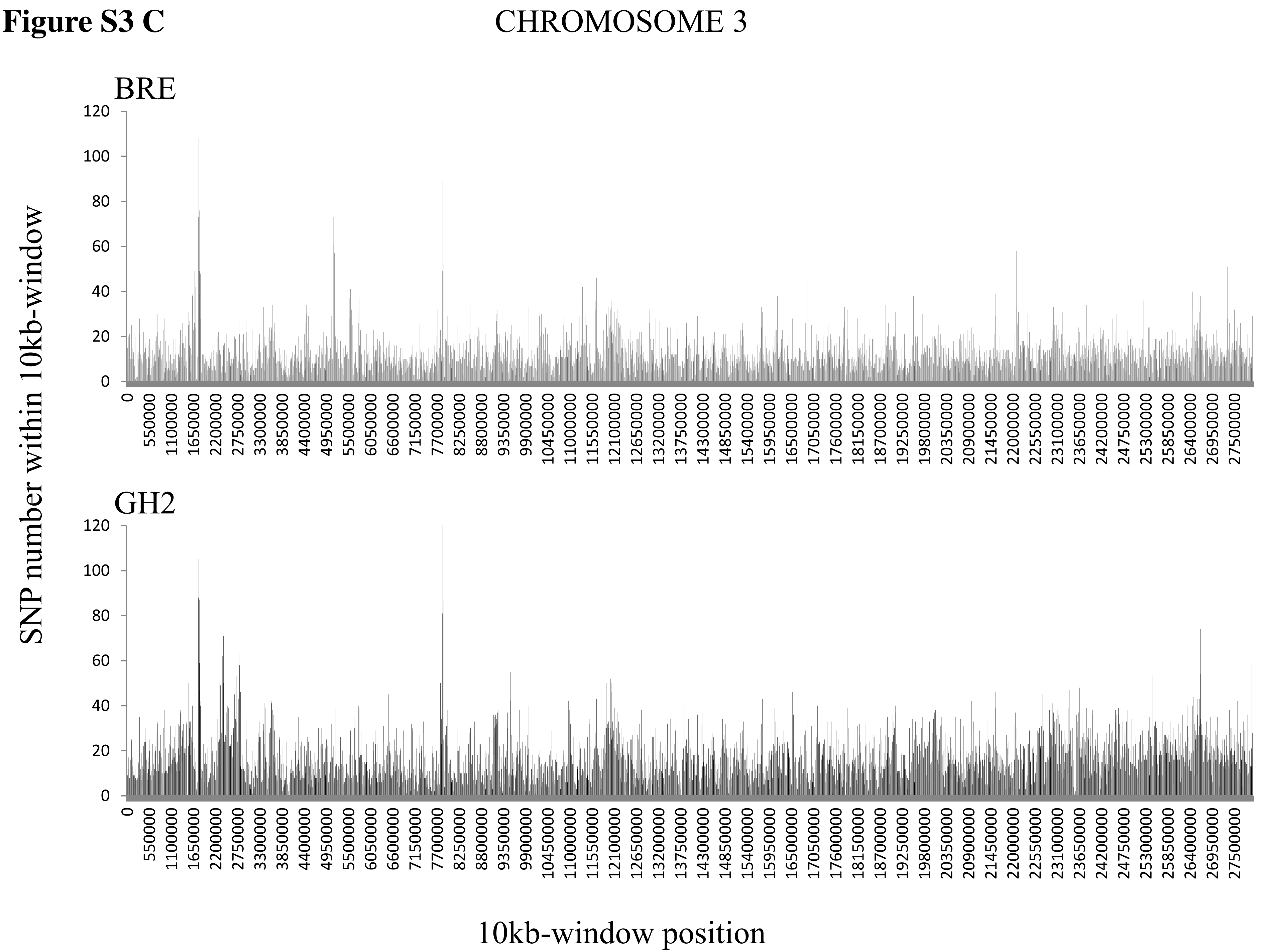
CHROMOSOME 3
BRE
GH2
SNP number within 10kb-window
10kb-window position
0 20 40 60 80 100 120
0 550000 1100000 1650000 2200000 2750000 3300000 3850000 4400000 4950000 5500000 6050000 6600000 7150000 7700000 8250000 8800000 9350000 9900000 10450000 11000000 11550000 12100000 12650000 13200000 13750000 14300000 14850000 15400000 15950000 16500000 17050000 17600000 18150000 18700000 19250000 19800000 20350000 20900000 21450000 22000000 22550000 23100000 23650000 24200000 24750000 25300000 25850000 26400000 26950000 27500000

Figure S3D

CHROMOSOME 4

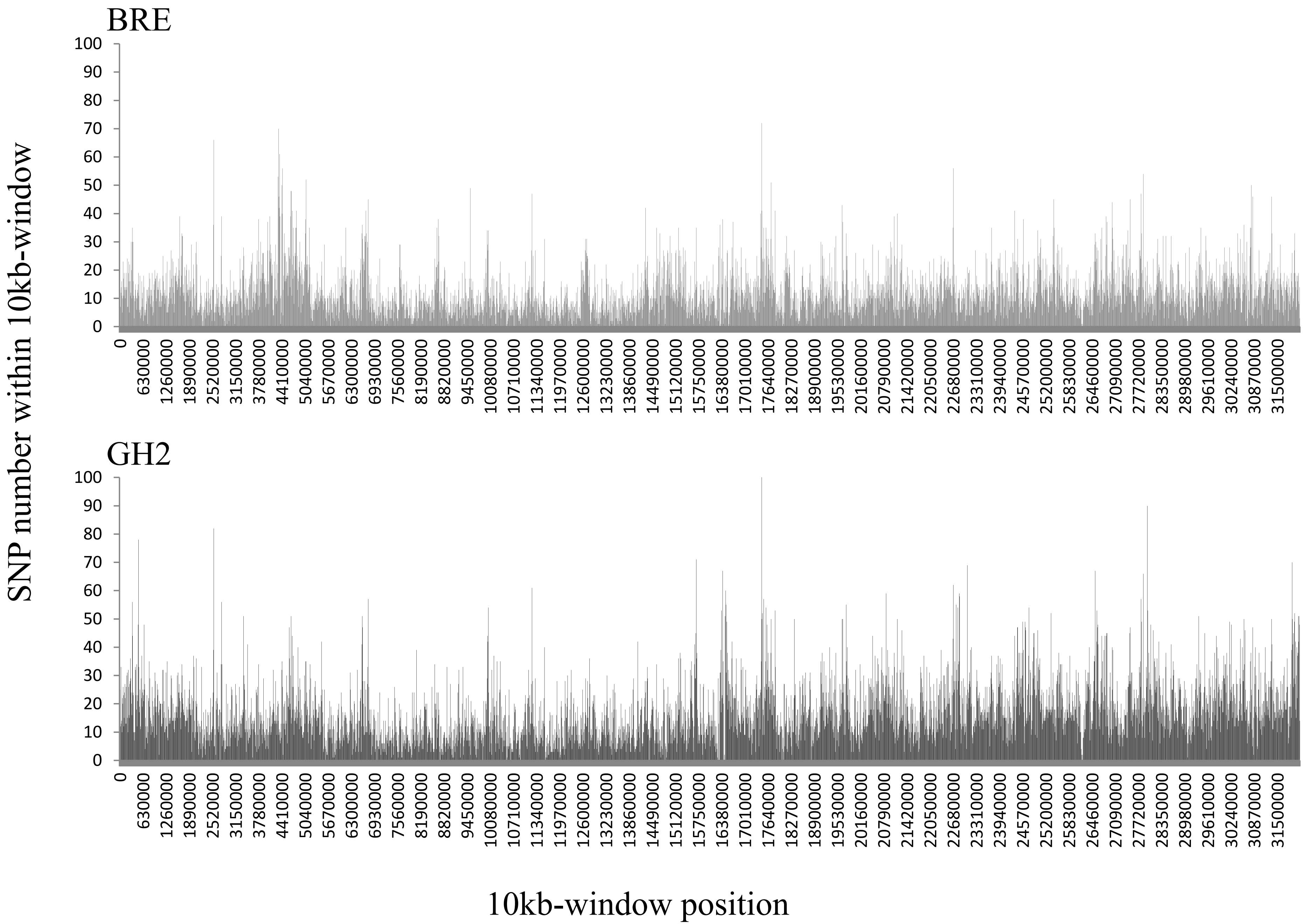

Figure S3E

CHROMOSOME 5

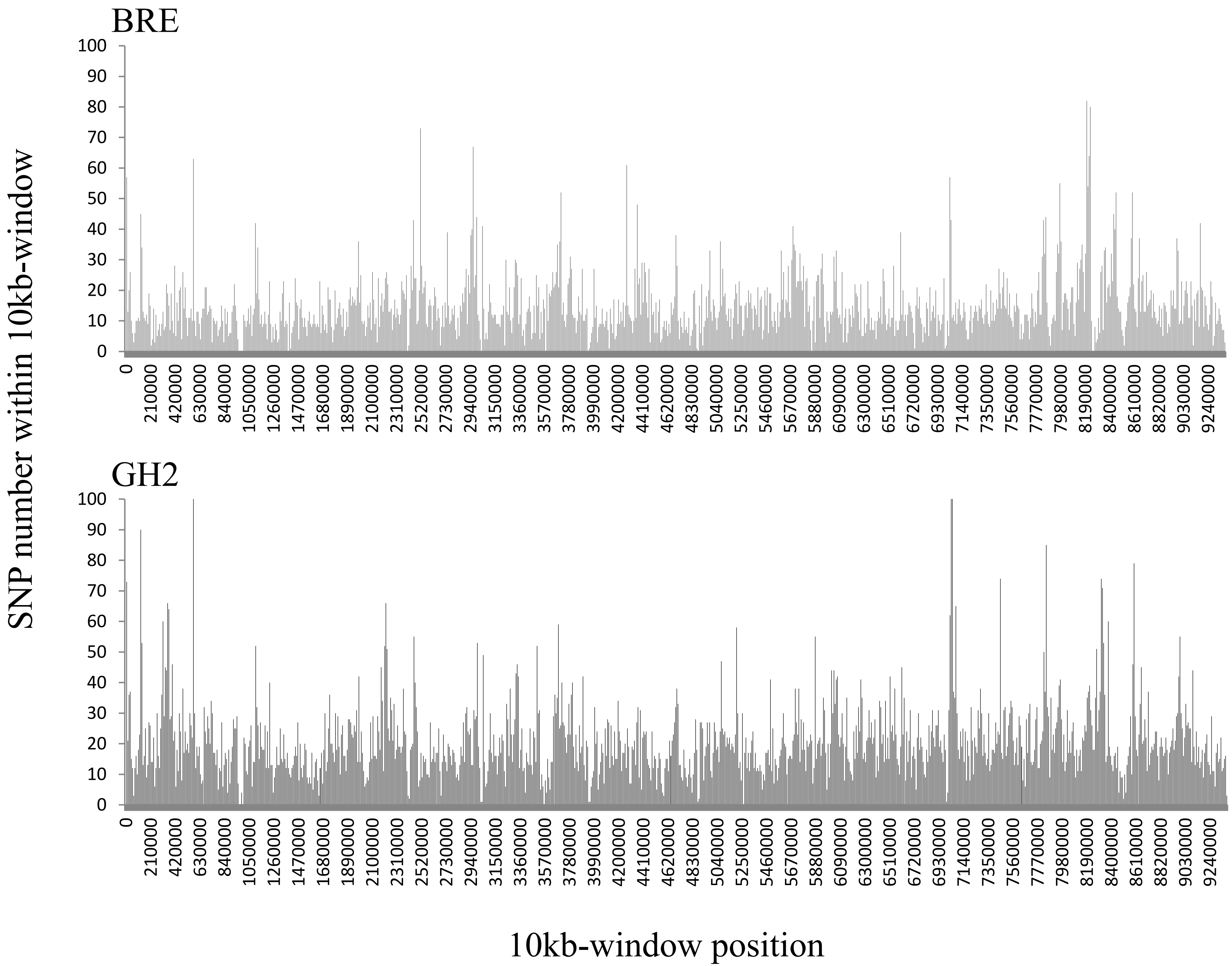

Figure S3 F

CHROMOSOME 6

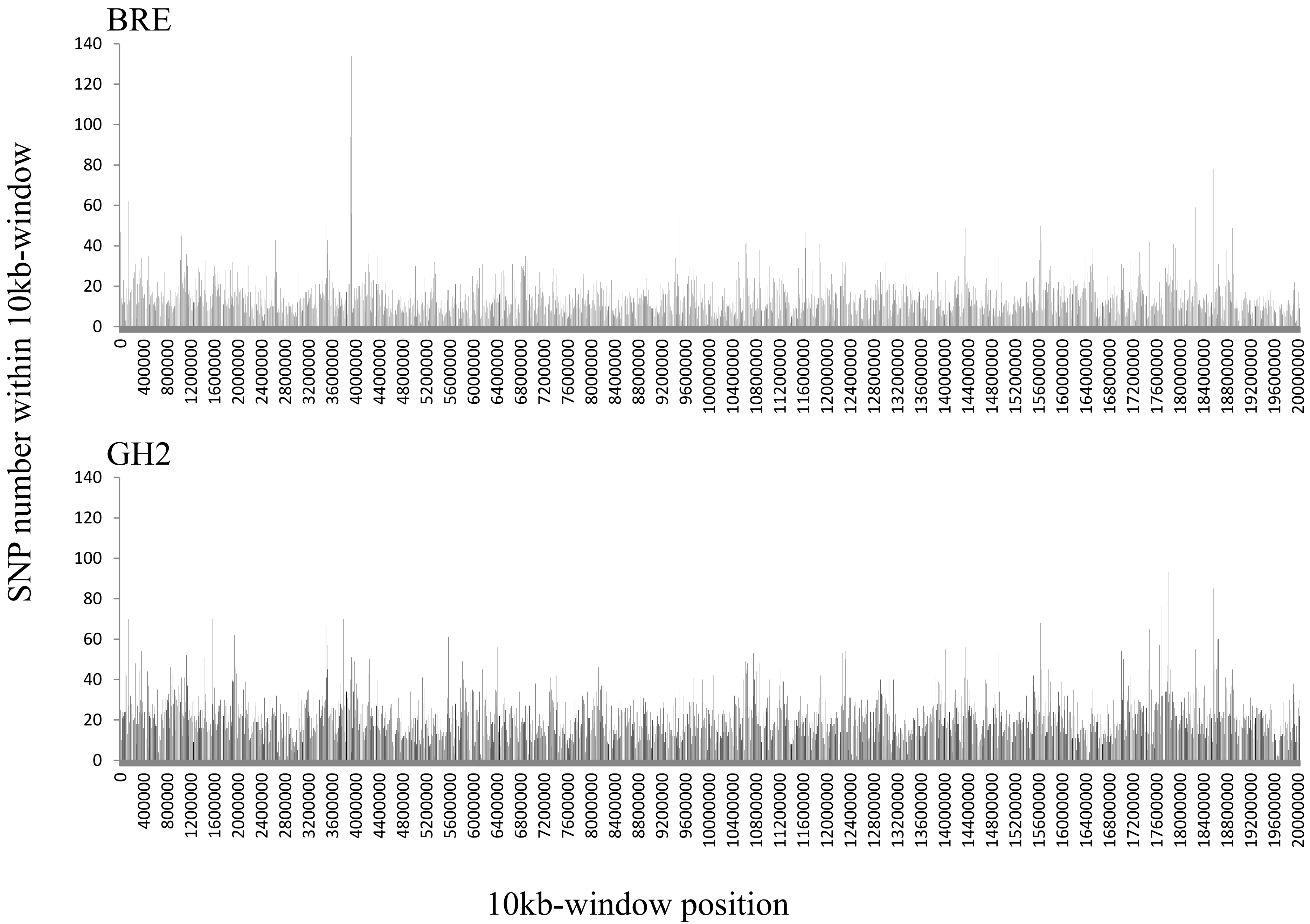

Figure S3G CHROMOSOME 7

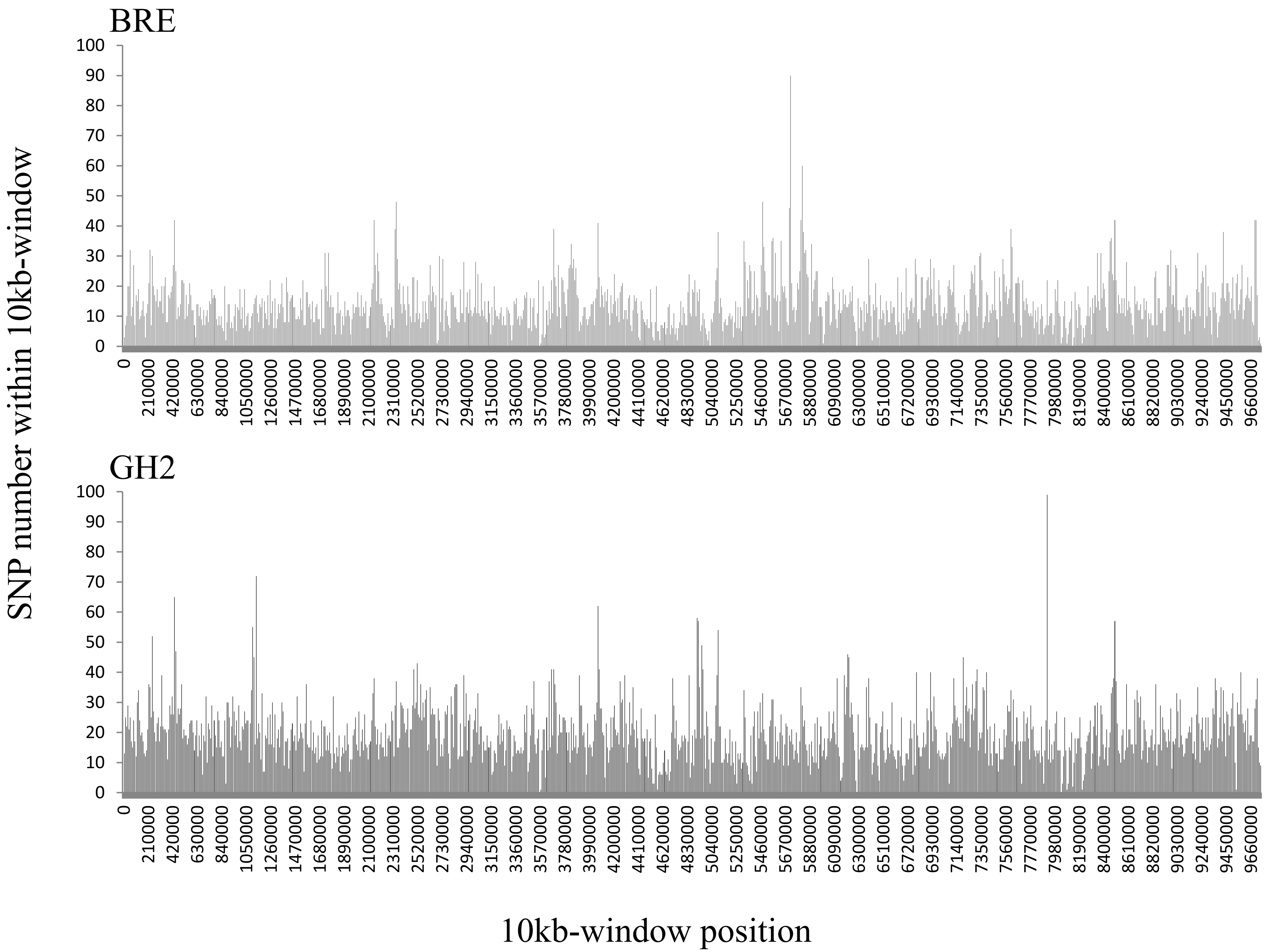

Figure S3 H

SEX CHROMOSOMES

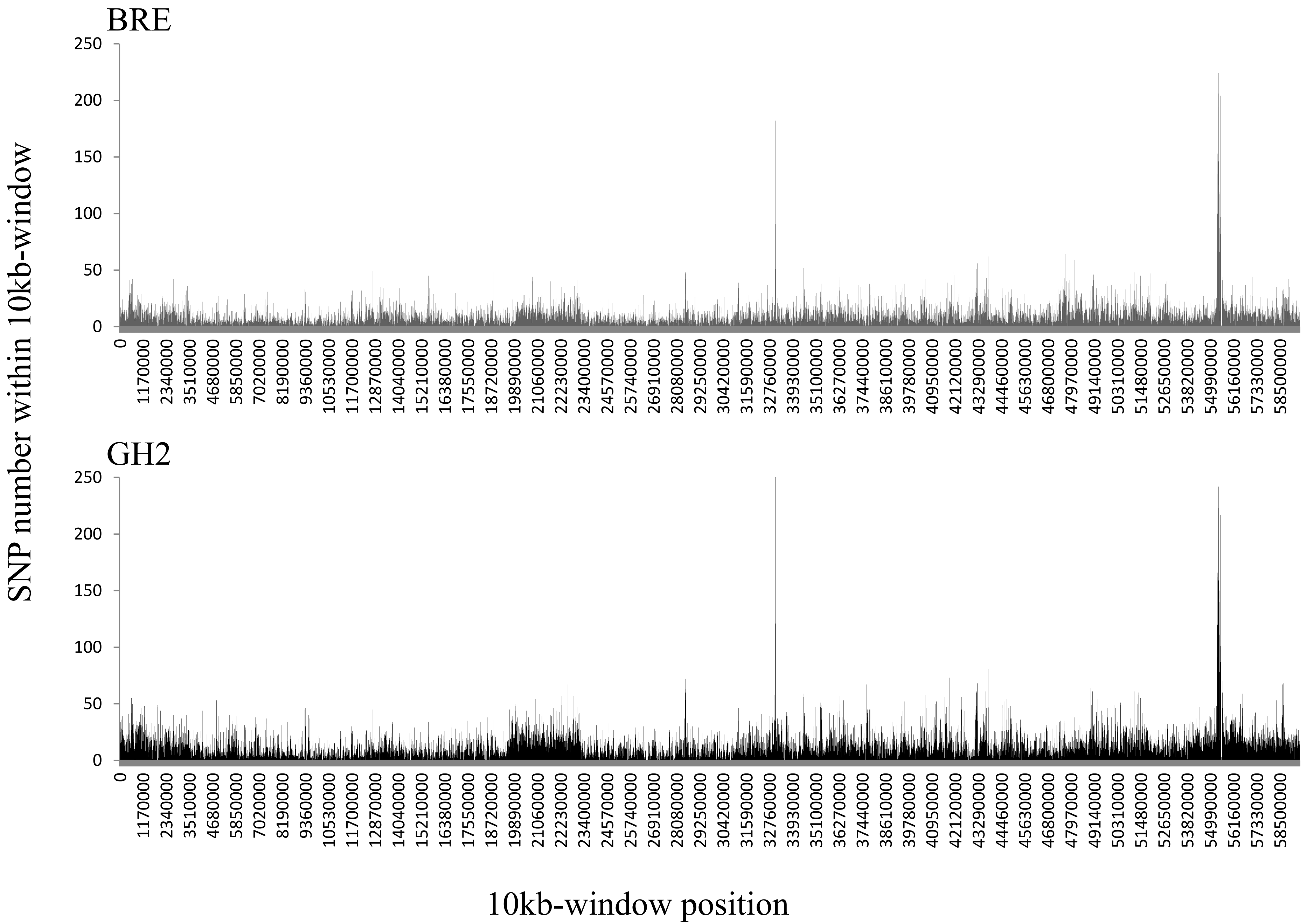

Supplement: Figure S3 — Distribution of SNP number in 10 kb-window across each of the seven autosomes (A to G) and across the ZW-linkage group (H) of Schistosoma mansoni. (PDF) [file pntd.0002591.s003.pdf]
