# Supplementary material for: Private Selective Sweeps Identified from Next-Generation Pool-Sequencing Reveal Convergent Pathways under Selection in Two Inbred Schistosoma mansoni Strains
Source: PLoS Negl Trop Dis. 2013 Dec 12;7(12):e2591. doi: 10.1371/journal.pntd.0002591 (PMC3861164; doi:10.1371/journal.pntd.0002591)
Supplement: Table S1 — Number and proportion of reads conserved after each step of bioinformatic treatment of Brazilian (BRE) and Guadeloupean (GH2) strains of Schistosoma mansoni. (DOCX) [file pntd.0002591.s007.docx]

Table S1

Number and proportion of reads conserved after each step of bioinformatic treatment of Brazilian (BRE) and Guadeloupean (GH2) strains of *Schistosoma mansoni*.

|  | BRE | GH2 |
| --- | --- | --- |
| Raw Clusters | 540,196,370 | 508,893,394 |
| Reads after quality filters | 203,373,434 | 204,453,418 |
| Reads successfully aligned | 95,474,777 | 122,346,146 |
