# Supplementary material for: Private Selective Sweeps Identified from Next-Generation Pool-Sequencing Reveal Convergent Pathways under Selection in Two Inbred Schistosoma mansoni Strains
Source: PLoS Negl Trop Dis. 2013 Dec 12;7(12):e2591. doi: 10.1371/journal.pntd.0002591 (PMC3861164; doi:10.1371/journal.pntd.0002591)
Supplement: Table S4 — Protein-coding genes in the Top 20 CNV regions (by length) in Schistosoma mansoni BRE and GH2. (DOCX) [file pntd.0002591.s010.docx]

Table S4

Protein-coding genes in the Top 20 CNV regions (by length) in *Schistosoma mansoni* BRE and GH2.

| Systematic_name | Product | CNV_number | size (bp) | log2 BRE/GH2 | chr_position |  |
| --- | --- | --- | --- | --- | --- | --- |
| Smp_148200 | junctophilin 2 | CNVR_38 | 268393 | 2.616539 | Schisto_mansoni.Chr_W:19678559..19946951 |  |
| Smp_048660 | nucleolar protein 56 | CNVR_38 |  |  | Schisto_mansoni.Chr_W:19678559..19946951 | |
| Smp_048650 | Histidine triad nucleotide binding protein 1 | CNVR_38 |  |  | Schisto_mansoni.Chr_W:19678559..19946951 | |
| Smp_148160 | Spermatogenesis associated protein 6 | CNVR_38 |  |  | Schisto_mansoni.Chr_W:19678559..19946951 | |
| Smp_048620 | exosome complex component RRP45 | CNVR_38 |  |  | Schisto_mansoni.Chr_W:19678559..19946951 | |
| Smp_148170 | hypothetical protein | CNVR_38 |  |  | Schisto_mansoni.Chr_W:19678559..19946951 | |
| Smp_148180 | hypothetical protein | CNVR_38 |  |  | Schisto_mansoni.Chr_W:19678559..19946951 | |
| Smp_148190 | transcription factor 7 2 | CNVR_38 |  |  | Schisto_mansoni.Chr_W:19678559..19946951 | |
| Smp_157980 | telomerase protein component 1 | CNVR_652 | 87025 | -1.472238 | Schisto_mansoni.Chr_3:1611110..1698134 | |
| Smp_066290 | telomerase protein component 1 | CNVR_652 |  |  | Schisto_mansoni.Chr_3:1611110..1698134 | |
| Smp_126550 | hypothetical protein | CNVR_652 |  |  | Schisto_mansoni.Chr_3:1611110..1698134 | |
| Smp_047550 | MORN repeat containing protein 3 | CNVR_18 | 68377 | 1.072369 | Schisto_mansoni.Chr_2:8614988..8683364 | |
| Smp_211100 (previously known as : Smp_047560) | inner nuclear membrane protein man1 | CNVR_18 |  |  | Schisto_mansoni.Chr_2:8614988..8683364 | |
| Smp_211090 (previously known as : Smp_047560) | peptide chain release factor 1 | CNVR_18 |  |  | Schisto_mansoni.Chr_2:8614988..8683364 | |
| Smp_047570 | hypothetical protein | CNVR_18 |  |  | Schisto_mansoni.Chr_2:8614988..8683364 | |
| Smp_047540 | f box and wd40 domain protein | CNVR_18 |  |  | Schisto_mansoni.Chr_2:8614988..8683364 | |
| Smp_148160 | Spermatogenesis associated protein 6 | CNVR_39 | 68177 | 1.978985 | Schisto_mansoni.Chr_W:19947815..20015991 | |
| Smp_148150 | Double strand break repair protein rad21 | CNVR_39 |  |  | Schisto_mansoni.Chr_W:19947815..20015991 | |
| Smp_202860 | hypothetical protein | CNVR_483 | 67599 | 2.809303 | Schisto_mansoni.Chr_5:8238920..8306518 | |
| Smp_136830 | subfamily A1A unassigned peptidase (A01 family) | CNVR_773 | 62937 | -3.934451 | Schisto_mansoni.Chr_3:23510078..23573014 | |
| Smp_136720 | subfamily A1A unassigned peptidase (A01 family) | CNVR_773 |  |  | Schisto_mansoni.Chr_3:23510078..23573014 | |
| Smp_136840 | subfamily A1A unassigned peptidase (A01 family) | CNVR_773 |  |  | Schisto_mansoni.Chr_3:23510078..23573014 | |
| Smp_173160 | family M13 unassigned peptidase (M13 family) | CNVR_270 | 58275 | -5.404273 | Schisto_mansoni.Chr_2:30215588..30273862 | |
| Smp_133830 | hypothetical protein | CNVR_116 | 56095 | -2.325448 | Schisto_mansoni.Chr_W:734845..790939 | |
| Smp_171770 | hypothetical protein | CNVR_828 | 47397 | 2.676763 | Schisto_mansoni.Chr_4:16540388..16587784 | |
| Smp_171780 | SPARC protein | CNVR_828 |  |  | Schisto_mansoni.Chr_4:16540388..16587784 | |
| Smp_192140 | NAD dependent epimerase:dehydratase | CNVR_368 | 41959 | -2.140441 | Schisto_mansoni.Chr_6:1533410..1575368 | |
| Smp_089330 | NAD dependent epimerase:dehydratase | CNVR_368 |  |  | Schisto_mansoni.Chr_6:1533410..1575368 | |
| Smp_131390 | PH interacting protein | CNVR_429 | 38849 | -2.005844 | Schisto_mansoni.Chr_6:17545050..17583898 | |
| Smp_016870 | protein regulator of cytokinesis 1 | CNVR_429 |  |  | Schisto_mansoni.Chr_6:17545050..17583898 | |
| Smp_032690 | hypothetical protein | CNVR_119 | 38073 | -1.488619 | Schisto_mansoni.Chr_2:716006..754078 | |
| Smp_032710 | hypothetical protein | CNVR_119 |  |  | Schisto_mansoni.Chr_2:716006..754078 | |
| Smp_128500 | lysine specific demethylase 8 | CNVR_43 | 37109 | 1.965735 | Schisto_mansoni.Chr_W:20764213..20801321 | |
| Smp_084450 | hypothetical protein | CNVR_43 |  |  | Schisto_mansoni.Chr_W:20764213..20801321 | |
| Smp_084460 | ribosomal protein L28 | CNVR_43 |  |  | Schisto_mansoni.Chr_W:20764213..20801321 | |
| Smp_084440 | hypothetical protein | CNVR_43 |  |  | Schisto_mansoni.Chr_W:20764213..20801321 | |
| Smp_179030 | hypothetical protein | CNVR_280 | 36247 | -1.343191 | Schisto_mansoni.Chr_W:16993765..17030011 | |
| Smp_179020 | hypothetical protein | CNVR_280 |  |  | Schisto_mansoni.Chr_W:16993765..17030011 | |
| Smp_124710 | hypothetical protein | CNVR_62 | 35743 | 2.816848 | Schisto_mansoni.Chr_2:26106812..26142554 | |
| Smp_004780 | immunophilin | CNVR_62 |  |  | Schisto_mansoni.Chr_2:26106812..26142554 | |
| Smp_137930 | hypothetical protein | CNVR_608 | 34189 | 1.706181 | Schisto_mansoni.Chr_3:5342264..5376452 | |
| Smp_192050 | egg protein CP391B | CNVR_1473 | 33411 | -4.549613 | Schisto_mansoni.Chr_1:65430006..65463416 | |
| Smp_153250 | Mediator of RNA polymerase II transcription | CNVR_14 | 32793 | 2.854577 | Schisto_mansoni.Chr_W:3326435..3359227 | |
