# Supplementary material for: Private Selective Sweeps Identified from Next-Generation Pool-Sequencing Reveal Convergent Pathways under Selection in Two Inbred Schistosoma mansoni Strains
Source: PLoS Negl Trop Dis. 2013 Dec 12;7(12):e2591. doi: 10.1371/journal.pntd.0002591 (PMC3861164; doi:10.1371/journal.pntd.0002591)
Supplement: Table S5 — Protein-coding genes in the Top 20 CNV regions (by log2ratio) in Schistosoma mansoni strains BRE and GH2. (DOCX) [file pntd.0002591.s011.docx]

Table S5

Protein-coding genes in the Top 20 CNV regions (by log2ratio ) in *Schistosoma mansoni* strains BRE and GH2.

| Systematic_name | Product | CNV_number | size (bp) | log2 BRE/GH2 | chr_position |
| --- | --- | --- | --- | --- | --- |
| Smp_084340 | ribosomal pseudouridine synthase | CNVR_271 | 21757 | -6,70223 | Schisto_mansoni.Chr_2:30274640..30296396 |
| Smp_091750 | hypothetical protein | CNVR_377 | 5439 | -6,576607 | Schisto_mansoni.Chr_6:3942888..3948326 |
| Smp_153370 | hypothetical protein | CNVR_448 | 5439 | 5,566192 | Schisto_mansoni.Chr_5:2399766..2405204 |
| Smp_173160 | family M13 unassigned peptidase (M13 family) | CNVR_270 | 58275 | -5,404273 | Schisto_mansoni.Chr_2:30215588..30273862 |
| Smp_191970 | CAI 2 protein | CNVR_417 | 6993 | -6,405771 | Schisto_mansoni.Chr_6:12364014..12371006 |
| Smp_199430 | nephrocystin 4 | CNVR_145 |  |  | Schisto_mansoni.Chr_2:8237366..8240474 |
| Smp_147660 | hypothetical protein | CNVR_145 | 3109 | -5,464218 | Schisto_mansoni.Chr_2:8237366..8240474 |
| Smp_133890 | hypothetical protein | CNVR_1 | 3453 | 4,629796 | Schisto_mansoni.Chr_W:462137..465589 |
| Smp_202140 | hypothetical protein | CNVR_634 | 3109 | 4,837445 | Schisto_mansoni.Chr_3:18412958..18416066 |
