# Supplementary material for: Private Selective Sweeps Identified from Next-Generation Pool-Sequencing Reveal Convergent Pathways under Selection in Two Inbred Schistosoma mansoni Strains
Source: PLoS Negl Trop Dis. 2013 Dec 12;7(12):e2591. doi: 10.1371/journal.pntd.0002591 (PMC3861164; doi:10.1371/journal.pntd.0002591)
Supplement: Table S7 — Common and specific pathways in which enzymes encoded by genes in genomic regions under selection were found for BRE and GH2 strains. (DOCX) [file pntd.0002591.s013.docx]

Table S7

Common and specific pathways in which enzymes encoded by genes in genomic regions under selection were found for BRE and GH2 strains.

| **BRE** | | **GH2** | |
| --- | --- | --- | --- |
| **Number of enzymes** | **Pathways** | | **Number of enzymes** |
| 2 | Purine metabolism | | 5 |
| 1 | Riboflavin metabolism | | 1 |
| 1 | Butanoate metabolism | | 1 |
| 1 | Aminobenzoate degradation | | 1 |
| 1 | Carbon fixation pathways in prokaryotes | | 1 |
| 1 | N-Glycan biosynthesis | Thiamine metabolism | 1 |
| 1 | Various types of N-glycan biosynthesis | Nicotinate and nicotinamide metabolism | 3 |
| 1 | Terpenoid backbone biosynthesis | Sulfur metabolism | 3 |
| 2 | Glycerolipid metabolism | Glycolysis / Gluconeogenesis | 2 |
| 1 | Cutin, suberine and wax biosynthesis | Pyruvate metabolism | 2 |
| 1 | Alanine, aspartate and glutamate metabolism | Selenocompound metabolism | 1 |
| 1 | Fructose and mannose metabolism | Carbon fixation in photosynthetic organisms | 1 |
| 1 | Amino sugar and nucleotide sugar metabolism | Fatty acid biosynthesis | 2 |
| 1 | Primary bile acid biosynthesis | Phosphatidylinositol signaling system | 2 |
| 1 | Fatty acid elongation | Tetracycline biosynthesis | 1 |
| 1 | Caprolactam degradation | Citrate cycle (TCA cycle) | 1 |
| 1 | Lysine degradation | Propanoate metabolism | 1 |
| 1 | Folate biosynthesis | Pyrimidine metabolism | 1 |
| 1 | Toluene degradation | Fatty acid elongation | 1 |
| 1 | Valine, leucine and isoleucine degradation | Inositol phosphate metabolism | 1 |
| 1 | Geraniol degradation | Glycerophospholipid metabolism | 1 |
| 1 | Zeatin biosynthesis | Biosynthesis of unsaturated fatty acids | 1 |
| 1 | Tryptophan metabolism |  |  |
| 1 | Fatty acid metabolism |  |  |
