# Supplementary material for: Private Selective Sweeps Identified from Next-Generation Pool-Sequencing Reveal Convergent Pathways under Selection in Two Inbred Schistosoma mansoni Strains
Source: PLoS Negl Trop Dis. 2013 Dec 12;7(12):e2591. doi: 10.1371/journal.pntd.0002591 (PMC3861164; doi:10.1371/journal.pntd.0002591)
Supplement: Table S8 — Primers used for the polymerase chain reaction (PCR) to check in situ 22 single nucleotide polymorphisms of both Brazilian and Guadeloupean strains of Schistosoma mansoni used in this study. (DOCX) [file pntd.0002591.s014.docx]

Table S8

Primers used for the polymerase chain reaction (PCR) to check *in situ* 22 single nucleotide polymorphisms of both Brazilian and Guadeloupean strains of *Schistosoma mansoni* used in this study.

| Name | Forward Primer (5'-3') | Reverse Primer (5'-3') | Product length | Number of SNPs within PCR product | Melting temperature |
| --- | --- | --- | --- | --- | --- |
| SmBreChr_1 | CAGCACAACTAACCTTTCTCCA | CAGCAGCAAACATTTTCAGG | 306 | 1 | 58°C |
| SmBreChr_2 | GTGAACGCCTGGTTCCTCTA | CGTGTTCGTCACCGAGTTTA | 443 | 1 | 59°C |
| SmBreChr_3 | GCGACCCTAAAGTCAAGCTG | GTTTGTCCCGTAGACCTCCA | 550 | 4 | 61°C |
| SmBreChr_4 | AATGGGGCTATGTCATCTGC | GCTGGCTTTAGACCTCATCG | 432 | 1 | 62°C |
| SmBreChr_5 | TTTGAATGGTCTTGGGCTTC | TGGGATTTGGATGGACAACT | 373 | 1 | 60°C |
| SmBreChr_7 | TCTGAGTTCCTGGCTTCTCG | CCCCAGATGCCTTAATGTTG | 454 | 1 | 62°C |
| SmGH2Chr_1.1 | TGACTCAGAGAATTCCGATGG | GATTGTTGTGGGGTGTTTCC | 648 | 2 | 58°C |
| SmGH2Chr_1.2 | GAATCGAAGCACTGTTCCAGA | GCATTTTCGGTGGAGGTAAA | 449 | 1 | 58°C |
| SmGH2Chr_2 | GTGTACCAACATCAACATAGATCG | GGAAGGGGAAGCTAACCATC | 525 | 2 | 60°C |
| SmGH2Chr_3 | GGGACTCACACACAGAACCA | GCATTCAAAGCGCAGAAGAT | 430 | 2 | 58°C |
| SmGH2Chr_5 | TGATCCCGCCTCACTAGATT | AGAAGGTGCCCACTGAAGAA | 519 | 3 | 59°C |
| SmGH2Chr_6 | AGGTAATCCCGTCAGATCCA | GACCATGTGGAAGCTGGAAT | 468 | 1 | 59°C |
| SmGH2Chr_7 | ATCAATTCGGCAACCACTGT | AAGTTGCGGCCTGACTACTG | 359 | 1 | 58°C |
| SmGH2Chr_W2 | GCGTCATTTTTGTACGACGA | CGTGAACCTGAAAACGCTTA | 531 | 1 | 58°C |
