# Supplementary material for: Private Selective Sweeps Identified from Next-Generation Pool-Sequencing Reveal Convergent Pathways under Selection in Two Inbred Schistosoma mansoni Strains
Source: PLoS Negl Trop Dis. 2013 Dec 12;7(12):e2591. doi: 10.1371/journal.pntd.0002591 (PMC3861164; doi:10.1371/journal.pntd.0002591)
Supplement: Table S9 — Number of selective sweeps detected on autosomes of Schistosoma mansoni strains BRE and GH2 used in this study for two values of transition probability q. (DOCX) [file pntd.0002591.s015.docx]

Table S9

Number of selective sweeps detected on autosomes of *Schistosoma mansoni* strains BRE and GH2 used in this study for two values of transition probability *q*.

|  | BRE | | GH2 | |
| --- | --- | --- | --- | --- |
| Chromosome | *q* = 10^-9^ | *q* = 10^-10^ | *q* = 10^-9^ | *q* = 10^-10^ |
| Chr1 | 54 | 37 | 63 | 50 |
| Chr2 | 31 | 25 | 39 | 26 |
| Chr3 | 26 | 17 | 28 | 19 |
| Chr4 | 22 | 14 | 36 | 20 |
| Chr5 | 16 | 9 | 12 | 8 |
| Chr6 | 18 | 12 | 25 | 19 |
| Chr7 | 11 | 7 | 11 | 9 |
| **Total** | **178** | **121** | **214** | **151** |
